# Supplementary material for: The relationship between sleep disorders and postoperative delirium in adult patients: Protocol for an updated systematic review and meta-analysis
Source: PLoS One. 2025 Dec 2;20(12):e0337598. doi: 10.1371/journal.pone.0337598 (PMC12671798; doi:10.1371/journal.pone.0337598)
Supplement: S2 File — (DOCX) [file pone.0337598.s002.docx]

**S2 File. Search strategies for included databases**

| **PubMed search details:**  1# "Delirium"[Mesh]  2# ((((((((Delirium[Title/Abstract]) OR (Delirium, Subacute[Title/Abstract])) OR (Deliriums, Subacute[Title/Abstract])) OR (Subacute Deliriums[Title/Abstract])) OR (Delirium of Mixed Origin[Title/Abstract])) OR (Mixed Origin Delirium[Title/Abstract])) OR (Mixed Origin Deliriums[Title/Abstract]) OR (Confusion[Title/Abstract])) OR (Emergence Agitation[Title/Abstract]))  3# 1# or 2#  4# "Postoperative Period"[Mesh]  5# "postoperative"[Title/Abstract] OR "postop*"[Title/Abstract] OR "post surg*"[Title/Abstract] OR "after surg*"[Title/Abstract] OR "surg*"[Title/Abstract] OR "anesthesia*"[Title/Abstract]  6# 4# or 5#  7# "Sleep Initiation and Maintenance Disorders"[Mesh] OR "Sleep Quality"[Mesh] OR "Sleep Wake Disorders"[Mesh] OR "Sleep"[Mesh]  8# (((((((((((((((((((((Sleeping Habits[Title/Abstract]) OR (Sleep Habits[Title/Abstract])) OR (Habit, Sleep[Title/Abstract])) OR (Habits, Sleep[Title/Abstract])) OR (Sleep Habit[Title/Abstract])) OR (Sleeping Habit[Title/Abstract])) OR (Habit, Sleeping[Title/Abstract])) OR (Habits, Sleeping[Title/Abstract])) OR (Sleep Initiation[Title/Abstract] AND Maintenance Disorders[Title/Abstract])) OR (Sleeplessness[Title/Abstract])) OR (Sleeplessness[Title/Abstract])) OR (Insomnia[Title/Abstract])) OR (Sleep Quality[Title/Abstract])) OR (Sleep Quality[Title/Abstract])) OR (Sleep Qualities[Title/Abstract])) OR (Disorder, Sleep Wake[Title/Abstract])) OR (Disorders, Sleep Wake[Title/Abstract])) OR (Sleep Wake Disorder[Title/Abstract])) OR (Wake Disorder, Sleep[Title/Abstract])) OR (Wake Disorders, Sleep[Title/Abstract])) OR (Sleep Disorders[Title/Abstract])) OR (Sleep Disorder[Title/Abstract])  9# 7# or #8  10# 3# and 6# and 9#  **Embase search details:**  1# 'delirium'/exp  2# 'acute delirium':ab,ti OR 'chronic delirium':ab,ti OR 'delier':ab,ti OR 'delire':ab,ti OR 'deliria':ab,ti OR 'delirious manifestation':ab,ti OR 'delirious state':ab,ti OR 'delirious syndrome':ab,ti OR 'delirium acutum':ab,ti OR 'delirium':ab,ti OR 'Confusion':ab,ti OR ' Emergence Agitation':ab,ti  3# 1# or 2#  4# 'postoperative period'/exp  5# 'post-operative period':ab,ti OR 'post-operative phase':ab,ti OR 'post-surgery period':ab,ti OR 'post-surgical period':ab,ti OR 'postoperative phase':ab,ti OR 'postsurgery period':ab,ti OR 'postsurgical period':ab,ti OR 'postoperative period':ab,ti OR 'post surgery':ab,ti OR 'surg*':ab,ti OR 'anesthesia*':ab,ti  6# 4# or 5#  7# 'sleep'/exp  8# 'sleeping':ab,ti OR 'sleep':ab,ti  9# 'sleep disorder'/exp  10# 'atypical sleep':ab,ti OR 'difficult sleeping':ab,ti OR 'difficulties in sleeping':ab,ti OR 'difficulty sleeping':ab,ti OR 'disorder during sleep':ab,ti OR 'disorder sleep':ab,ti OR 'disorders during sleep':ab,ti OR 'disorders sleep':ab,ti OR 'disturbance during sleep':ab,ti OR 'disturbance of sleep':ab,ti OR 'disturbances during sleep':ab,ti OR 'disturbances of sleep':ab,ti OR 'problem during sleep':ab,ti OR 'problem of sleep':ab,ti OR 'problems during sleep':ab,ti OR 'problems of sleep':ab,ti OR 'sleep disorders':ab,ti OR 'sleep disturbance':ab,ti OR 'sleep disturbances':ab,ti OR 'sleep interference':ab,ti OR 'sleep perturbation':ab,ti OR 'sleep perturbations':ab,ti OR 'sleep problem':ab,ti OR 'sleep problems':ab,ti OR 'sleep related disease':ab,ti OR 'sleep related disorder':ab,ti OR 'sleep related problem':ab,ti OR 'sleep wake disorder':ab,ti OR 'sleep wake disorders':ab,ti OR 'sleeping difficulties':ab,ti OR 'sleeping difficulty':ab,ti OR 'sleeping disorder':ab,ti OR 'sleeping disorders:ab,ti' OR 'sleeping problem':ab,ti OR 'sleeping problems':ab,ti OR 'trouble sleeping':ab,ti OR 'sleep disorder':ab,ti  11# 'sleep quality'/exp  12# 'sleep quality':ab,ti  13# 'insomnia'/exp  14# 'agrypnia':ab,ti OR 'disorder of sleep initiation and maintenance':ab,ti OR 'disorders of sleep initiation and maintenance':ab,ti OR 'hyposomnia':ab,ti OR 'hyposomnias':ab,ti OR 'insomnia disorder':ab,ti OR 'insomnia disorders':ab,ti OR 'insomnias':ab,ti OR 'sleep initiation and maintenance disorder':ab,ti OR 'sleep initiation and maintenance disorders':ab,ti OR 'sleep initiation/maintenance disorder':ab,ti OR 'sleeplessness':ab,ti OR 'insomnia':ab,ti  15# #7 OR #8 OR #9 OR #10 OR #11 OR #12 OR #13 OR #14  16# 3# and 6# and 15#  **CINAHL search details:**  1# MH "Delirium"  2# TI (“Delirium” OR “Delirium, Subacute” OR “Deliriums, Subacute” OR “Subacute Deliriums” OR “Delirium of Mixed Origin” OR “Mixed Origin Delirium” OR “Mixed Origin Deliriums” OR “confusion” OR “emergence agitation”) OR AB (“Delirium” OR “Delirium, Subacute” OR “Deliriums, Subacute” OR “Subacute Deliriums” OR “Delirium of Mixed Origin” OR “Mixed Origin Delirium” OR “Mixed Origin Deliriums” OR “Confusion” OR “Emergence Agitation”)  3# 1# or 2#  4# MH "Postoperative Period"  5# TI ("postoperative" OR "postop*" OR "post surg*" OR "after surg*" OR "surg*" OR "anesthesia*") OR AB ("postoperative" OR "postop*" OR "post surg*" OR "after surg*" OR "surg*" OR " anesthesia*")  6# 4# or 5#  7# MH "Sleep Initiation and Maintenance Disorders" OR MH "Sleep Quality" OR MH "Sleep Wake Disorders" OR MH "Sleep"  8# TI (“Sleeping Habits” OR “Sleep Habits” OR “Habit, Sleep” OR “Habits, Sleep” OR “Sleep Habit” OR “Sleeping Habit” OR “Habit, Sleeping” OR “Habits, Sleeping” OR "Sleep Initiation” AND “Maintenance Disorders” OR “Sleeplessness” OR “Sleeplessness” OR “Insomnia” OR “Sleep Quality” OR “Sleep Quality” OR “Sleep Qualities” OR “Disorder, Sleep Wake” OR “Disorders, Sleep Wake” OR “Sleep Wake Disorder” OR “Wake Disorder, Sleep” OR “Wake Disorders, Sleep” OR “Sleep Disorders” OR “Sleep Disorder”) OR AB (“Sleeping Habits” OR “Sleep Habits” OR “Habit, Sleep” OR “Habits, Sleep” OR “Sleep Habit” OR “Sleeping Habit” OR “Habit, Sleeping” OR “Habits, Sleeping” OR "Sleep Initiation” AND “Maintenance Disorders” OR “Sleeplessness” OR “Sleeplessness” OR “Insomnia” OR “Sleep Quality” OR “Sleep Quality” OR “Sleep Qualities” OR “Disorder, Sleep Wake” OR “Disorders, Sleep Wake” OR “Sleep Wake Disorder” OR “Wake Disorder, Sleep” OR “Wake Disorders, Sleep” OR “Sleep Disorders” OR “Sleep Disorder”)  9# 7# or #8  10# 3# and 6# and 9#  **PsycINFO search details:**  1# SU "Delirium"  2# TI (“Delirium” OR “Delirium, Subacute” OR “Deliriums, Subacute” OR “Subacute Deliriums” OR “Delirium of Mixed Origin” OR “Mixed Origin Delirium” OR “Mixed Origin Deliriums” OR “confusion” OR “emergence agitation”) OR AB (“Delirium” OR “Delirium, Subacute” OR “Deliriums, Subacute” OR “Subacute Deliriums” OR “Delirium of Mixed Origin” OR “Mixed Origin Delirium” OR “Mixed Origin Deliriums” OR “Confusion” OR “Emergence Agitation”)  3# 1# or 2#  4# SU "Postoperative Period"  5# TI ("postoperative" OR "postop*" OR "post surg*" OR "after surg*" OR "surg*" OR "anesthesia*") OR AB ("postoperative" OR "postop*" OR "post surg*" OR "after surg*" OR "surg*" OR "anesthesia*")  6# 4# or 5#  7# SU "Sleep Initiation and Maintenance Disorders" OR SU "Sleep Quality" OR SU "Sleep Wake Disorders" OR SU "Sleep"  8# TI (“Sleeping Habits” OR “Sleep Habits” OR “Habit, Sleep” OR “Habits, Sleep” OR “Sleep Habit” OR “Sleeping Habit” OR “Habit, Sleeping” OR “Habits, Sleeping” OR "Sleep Initiation” AND “Maintenance Disorders” OR “Sleeplessness” OR “Sleeplessness” OR “Insomnia” OR “Sleep Quality” OR “Sleep Quality” OR “Sleep Qualities” OR “Disorder, Sleep Wake” OR “Disorders, Sleep Wake” OR “Sleep Wake Disorder” OR “Wake Disorder, Sleep” OR “Wake Disorders, Sleep” OR “Sleep Disorders” OR “Sleep Disorder”) OR AB (“Sleeping Habits” OR “Sleep Habits” OR “Habit, Sleep” OR “Habits, Sleep” OR “Sleep Habit” OR “Sleeping Habit” OR “Habit, Sleeping” OR “Habits, Sleeping” OR "Sleep Initiation” AND “Maintenance Disorders” OR “Sleeplessness” OR “Sleeplessness” OR “Insomnia” OR “Sleep Quality” OR “Sleep Quality” OR “Sleep Qualities” OR “Disorder, Sleep Wake” OR “Disorders, Sleep Wake” OR “Sleep Wake Disorder” OR “Wake Disorder, Sleep” OR “Wake Disorders, Sleep” OR “Sleep Disorders” OR “Sleep Disorder”)  9# 7# or #8  10# 3# and 6# and 9# |
| --- |
