# Supplementary material for: The relationship between sleep disorders and postoperative delirium in adult patients: Protocol for an updated systematic review and meta-analysis
Source: PLoS One. 2025 Dec 2;20(12):e0337598. doi: 10.1371/journal.pone.0337598 (PMC12671798; doi:10.1371/journal.pone.0337598)
Supplement: Attachment — Submitted filename: PONE-D-25-29095 fc.pdf [file pone.0337598.s003.pdf]

PLOS ONE

The relationship between sleep disorders and postoperative delirium in adult patients: protocol for an updated systematic review and meta-analysis

--Manuscript Draft--

**Manuscript Number:**	PONE-D-25-29095
**Article Type:**	Study Protocol
**Full Title:**	The relationship between sleep disorders and postoperative delirium in adult patients: protocol for an updated systematic review and meta-analysis
**Short Title:**	The relationship between sleep disorders and postoperative delirium in adult patients
**Corresponding Author:**	Wei Wei<br>West China Hospital of Sichuan University<br>Chengdu, CHINA
**Keywords:**	Sleep disorders; postoperative delirium; Adult; Meta-analysis
**Abstract:**	Introduction: Postoperative delirium (POD) is a common complication after surgery. The association between sleep disorders and the risk of POD has been widely studied. Sleep disorders have emerged as a potential risk factor for POD, but recent studies provide conflicting evidence regarding the existence and the extent of the association. The aim of this systematic review and meta-analysis will be to estimate the association between sleep disorders and the risk of POD in adult patients.<br>Methods and analysis：A systematic review will be conducted to estimate the association between sleep disorders and the risk of POD in adult patients. This systematic review protocol follows the Preferred Reporting Items for Systematic Review and Meta-Analysis Protocols (PRISMA-P) statement. Literature searches will be carried out in the Pubmed, Embase, and Cochrane Library databases from inception until May 2025 without language restrictions. Only observational studies that investigated the association between sleep disorders and POD will be included. Two independent reviewers will be responsible for the selection of studies, extraction of information and risk of bias assessment of the articles. A random effects model will be used to calculate the pooled risk estimates for the outcome. Subgroup analysis will be used to explore potential sources of heterogeneity. Publication bias will be estimated by funnel plots and Egger's test. Sensitivity analysis will also be performed to explore the stability of the overall effect size. Also, evidence quality will be assessed. All data will be analysed using Review Manager (V.5.3) and Stata (V.14.0) statistical software.<br>Discussion: The proposed systematic review and meta-analysis will highlight the association of sleep disorders and the risk of POD in adult patients. The findings of this review and meta-analysis will help clinicians consider the sleep disorders to make better health decisions.
**Order of Authors:**	Qianli Huang
	Changhui Shao
	Ling Peng
	Wei Wei
**Additional Information:**	
**Question**	**Response**
**Financial Disclosure**<br><br>Enter a financial disclosure statement that describes the sources of funding for the work included in this submission. Review the submission guidelines for detailed requirements. View published research articles from *PLOS ONE* for specific	Yes

Powered by Editorial Manager® and ProduXion Manager® from Aries Systems Corporation

<table>
<tr><td>examples.

This statement is required for submission and will appear in the published article if the submission is accepted. Please make sure it is accurate.

Funded studies
Enter a statement with the following details:
• Initials of the authors who received each award
• Grant numbers awarded to each author
• The full name of each funder
• URL of each funder website
• Did the sponsors or funders play any role in the study design, data collection and analysis, decision to publish, or preparation of the manuscript?

Did you receive funding for this work?</td><td></td></tr>
<tr><td>Please add funding details.
as follow-up to "Financial Disclosure

Enter a financial disclosure statement that describes the sources of funding for the work included in this submission. Review the submission guidelines for detailed requirements. View published research articles from PLOS ONE for specific examples.

This statement is required for submission and will appear in the published article if the submission is accepted. Please make sure it is accurate.

Funded studies
Enter a statement with the following details:
• Initials of the authors who received each award
• Grant numbers awarded to each author
• The full name of each funder
• URL of each funder website
• Did the sponsors or funders play any role in the study design, data collection and analysis, decision to publish, or preparation of the manuscript?</td><td>The research work was funded by a grant from the National Natural Science Foundation of China (grant no. 81971772).</td></tr>
</table>

Powered by Editorial Manager® and ProduXion Manager® from Aries Systems Corporation

Did you receive funding for this work?"	
Please select the country of your main research funder (please select carefully as in some cases this is used in fee calculation).<br>as follow-up to "**Financial Disclosure**<br><br>Enter a financial disclosure statement that describes the sources of funding for the work included in this submission. Review the submission guidelines for detailed requirements. View published research articles from *PLOS ONE* for specific examples.<br><br>This statement is required for submission and **will appear in the published article** if the submission is accepted. Please make sure it is accurate.<br><br>**Funded studies**<br>Enter a statement with the following details:<br>• Initials of the authors who received each award<br>• Grant numbers awarded to each author<br>• The full name of each funder<br>• URL of each funder website<br>• Did the sponsors or funders play any role in the study design, data collection and analysis, decision to publish, or preparation of the manuscript?<br><br>Did you receive funding for this work?"	CHINA - CN
**Competing Interests**<br><br>Use the instructions below to enter a competing interest statement for this submission. On behalf of all authors, disclose any competing interests that could be perceived to bias this work—acknowledging all financial support and any other relevant financial or non-financial competing interests.	The authors have declared that no competing interests exist.

This statement is required for submission and **will appear in the published article** if the submission is accepted. Please make sure it is accurate and that any funding sources listed in your Funding Information later in the submission form are also declared in your Financial Disclosure statement.<br><br>View published research articles from *PLOS ONE* for specific examples.<br><br>**NO authors have competing interests**<br><br>Enter: *The authors have declared that no competing interests exist.*<br><br>**Authors with competing interests**<br><br>Enter competing interest details beginning with this statement:<br><br>*I have read the journal's policy and the authors of this manuscript have the following competing interests: [insert competing interests here]*<br><br>* typeset	
**Ethics Statement**<br><br>Enter an ethics statement for this submission. This statement is required if the study involved:<br><br>• Human participants<br>• Human specimens or tissue<br>• Vertebrate animals or cephalopods<br>• Vertebrate embryos or tissues<br>• Field research<br><br>Write "N/A" if the submission does not require an ethics statement.<br><br>General guidance is provided below. Consult the submission guidelines for detailed instructions. **Make sure that all information entered here is included in the Methods section of the manuscript.**	N/A

**Format for specific study types**<br><br>**Human Subject Research (involving human participants and/or tissue)**<br>• Give the name of the institutional review board or ethics committee that approved the study<br>• Include the approval number and/or a statement indicating approval of this research<br>• Indicate the form of consent obtained (written/oral) or the reason that consent was not obtained (e.g. the data were analyzed anonymously)<br><br>**Animal Research (involving vertebrate animals, embryos or tissues)**<br>• Provide the name of the Institutional Animal Care and Use Committee (IACUC) or other relevant ethics board that reviewed the study protocol, and indicate whether they approved this research or granted a formal waiver of ethical approval<br>• Include an approval number if one was obtained<br>• If the study involved *non-human primates*, add *additional details* about animal welfare and steps taken to ameliorate suffering<br>• If anesthesia, euthanasia, or any kind of animal sacrifice is part of the study, include briefly which substances and/or methods were applied<br><br>**Field Research**<br><br>Include the following details if this study involves the collection of plant, animal, or other materials from a natural setting:<br>• Field permit number<br>• Name of the institution or relevant body that granted permission	
**Data Availability**<br><br>Authors are required to make the data underlying their research findings fully available, without restriction.<br><br>If the manuscript reports pilot or preliminary results, the data underlying those results must be made available at	N/A - No results are reported

the time of publication. A Data Availability Statement describing where the data can be found is required at submission. Your answers to this question constitute the Data Availability Statement and will be published in the article, if accepted. When legal or ethical requirements prohibit public data sharing, authors must indicate how researchers may obtain access to the data. See the PLOS Data Policy and FAQ for detailed information.<br><br>**Important:** Stating 'data available on request from the author' is not sufficient. If your data are only available upon request, select 'No' for the first question and explain your situation in the text box.<br><br>For protocols without pilot or preliminary data, authors are strongly encouraged to state how they plan to share research data from their study when it is completed or published.<br><br>If your study includes pilot data, will they be made fully available without restriction?	
**Describe where the data may be found in full sentences. If you are copying our sample text, replace any instances of XXX with the appropriate details.**<br><br>• If **no pilot data are reported** in the manuscript, please enter the following: *No datasets were generated or analysed during the current study. All relevant data from this study will be made available upon study completion.*<br>• If the manuscript reports pilot data **held in a public repository**, include URLs, accession numbers or DOIs. If this information will only be available after acceptance, indicate this by ticking the box below. For example: *All XXX files are available from the XXX database (accession number(s) XXX, XXX.).*<br>• If the manuscript reports pilot data	All relevant data are within the manuscript and its Supporting Information files.

contained **within the manuscript and/or Supporting Information files**, enter the following:<br>*All relevant data are within the manuscript and its Supporting Information files.*<br>• If neither of these applies but you are able to provide **details of access elsewhere**, with or without limitations, please do so. For example:<br><br>*Data cannot be shared publicly because of [XXX]. Data are available from the XXX Institutional Data Access / Ethics Committee (contact via XXX) for researchers who meet the criteria for access to confidential data.*<br><br>*The data underlying the results presented in the study are available from (include the name of the third party and contact information or URL).*<br>• This text is appropriate if the data are owned by a third party and authors do not have permission to share the data.<br><br>For study protocols with data management or sharing plans, authors are encouraged to briefly describe in their Data Availability Statement how data they generate will be made accessible when the study is completed. For example:<br>• Deidentified research data will be made publicly available when the study is completed and published.<br><br>* typeset	
Additional data availability information:	

Powered by Editorial Manager® and ProduXion Manager® from Aries Systems Corporation

Manuscript Click here to access/download;Manuscript;Manuscript.docx

The relationship between sleep disorders and postoperative delirium in adult patients: protocol for an updated systematic review and meta-analysis

Qianli Huang[1 2#], Changhui Shao[3#], Ling Peng[1], Wei Wei[1*]

[1]Department of Anesthesiology, West China Hospital, Sichuan University, Chengdu, Sichuan, China.

[2]Department of Anesthesiology, Chengdu Women's and Children's Central Hospital, School of Medicine, University of Electronic Science and Technology of China, Chengdu, Sichuan, China.

[3]Department of Anesthesiology, Chengdu integrated TCM & Western Medicine Hospital, Chengdu, Sichuan, China.

*Corresponding author

[#] These authors contributed equally to this work

Emails of authors: 1172430737@qq.com (HQL); 814660661@qq.com (CHS); penglingjq@163.com (LP); weiw@scu.edu.cn (WW).

Abstract

Introduction: Postoperative delirium (POD) is a common complication after surgery. The association between sleep disorders and the risk of POD has been widely studied. Sleep disorders have emerged as a potential risk factor for POD, but recent studies provide conflicting evidence regarding the existence and the extent of the association. The aim of this systematic review and meta-analysis will be to estimate the association between sleep disorders and the risk of POD in adult patients.

Methods and analysis: A systematic review will be conducted to estimate the association between sleep disorders and the risk of POD in adult patients. This systematic review protocol follows the Preferred Reporting Items for Systematic Review and Meta-Analysis Protocols (PRISMA-P) statement. Literature searches will be carried out in the Pubmed, Embase, and Cochrane Library databases from inception until May 2025 without language restrictions. Only observational studies that investigated the association between sleep disorders and POD will be included. Two independent reviewers will be responsible for the selection of studies, extraction of information and risk of bias assessment of the articles. A random effects model will be used to calculate the pooled risk estimates for the outcome. Subgroup analysis will be used to explore potential sources of heterogeneity. Publication bias will be estimated by funnel plots and Egger's test. Sensitivity analysis will also be performed to explore the stability of the overall effect size. Also, evidence quality will be assessed. All data will be analysed using Review Manager (V.5.3) and Stata (V.14.0) statistical software.

Discussion: The proposed systematic review and meta-analysis will highlight the association of sleep disorders and the risk of POD in adult patients. The findings of this review

and meta-analysis will help clinicians consider the sleep disorders to make better health decisions.

Trial registration: Prospero registration number: CRD42024604118

Introduction

Postoperative delirium (POD) a is common and serious postoperative complication, affecting up to 50% of patients in some cohorts[1]. It is associated with a range of adverse outcomes, such as prolonged hospital stays, elevated healthcare costs, cognitive impairment, and higher risk of mortality and morbidity[2-4]. Given the significant adverse outcomes associated with POD, it is crucial for clinicians to identify risk factors and quantify their impact on POD.

Sleep is a complex physiological process that is essential for overall health and well-being. Sleep disorders are highly prevalent during the perioperative period, including conditions such as insomnia, sleep-disordered breathing, and circadian rhythm disruption. Recent studies have shown that 8.8%-79.1% of patients complained about poor sleep quality in the preoperative period[5-7]. Additionally, postoperative sleep disorders are highly prevalent in the first 2 weeks after surgery[8]. In the context of surgical patients, perioperative sleep disturbances may be associated with adverse prognostic outcomes, such as higher incidence of delirium and cognitive impairment, increased postoperative pain intensity, delayed postoperative recovery, increased hospital length of stay, and poor quality of life[9-11].

Extensive research has investigated the relationship between sleep disorders and the risk of POD. An earlier meta-analysis concluded that pre-operative sleep disturbances are likely associated with POD[12]. However, whether postoperative sleep disturbances serve as a risk factor for POD is still unknown. Initially, a meta-analysis conducted by Wang et al. had investigated association between perioperative sleep disturbance and the risk of POD[13]. Results showed that perioperative sleep disturbances were associated with an increased risk of POD in observational trials, yet no significant association was detected in randomized

controlled trials. Meanwhile, this study was limited by high heterogeneity and a small sample size, which may have affected the generalizability and statistical power of the findings. A recent meta-analysis incorporated 16 observational studies which concluded that sleep disturbance may increase the risk of POD[14]. This analysis offered valuable insights but was limited by relatively small sample sizes and the inclusion of only English-language literature.

However, three years have elapsed since the previous meta-analysis, and newly published trials with large-scale investigations may provide further insights[15, 16]. By synthesizing existing data, an updated systematic review will provide more robust guidance on the relationship between sleep disorders and the risk of POD.

Methods

Protocol and registration

This systematic review and meta-analysis was performed and written in accordance with the Preferred Reporting Items for Systematic Reviews and Meta-Analyses (PRISMA) guidelines[17] and the study protocol was registered in the International Prospective Register of Systematic Reviews (PROSPERO) (registration number: CRD42024604118). The PRISMA-P-checklist is shown in S1 File.

Ethics and dissemination

As this systematic review is summary and analysis of existing literature, there is no requirement for ethics approval. The results of the study will be published in a peer-reviewed journal.

Patient and public involvement

Patients and/or the public were not involved in the design, or conduct, or reporting, or dissemination plans of this research.

Objectives and outcomes

The sole objective of this is to determine whether perioperative sleep disorders are associated with an increased risk of POD in adult patients after surgery. The study aims to answer the research questions based on PECOS (Population, Exposure, Compares, Outcomes and Study design) criteria:

What is the association between sleep disorders and the risk of POD in adult patients after surgery?

PECOS eligibility criteria

- Population: adult patients aged 18 years and older undergoing surgery.
- Exposure: sleep disorders were used as the overarching term during the perioperative period.
- Comparator: the population without exposure to sleep disorders served as a comparator
- Outcome: publicly published research on the risk of POD with and without sleep disorders.
- Study design: the study type was observational studies including cohort study, cross-sectional study, or case–control study.

The inclusion criteria are as follows:

1. Studies investigating the association of sleep disorders and the risk of POD.
2. Included participants diagnosed with sleep disorders (insomnia symptoms, poor sleep quality, and obstructive sleep apnoea) and no sleep disorders.

The exclusion criteria are as follows:

1. Studies without measurement of sleep disorders or POD.
2. Review, meta-analyses, letter, editorial, protocol, case reports, or animal experiments.

Information sources

We will conduct searches in the following databases, including PubMed, Embase, and Cochrane Library, for studies published before May 2025, that reported on the association between sleep disorders and postoperative delirium in adult patients. No restrictions will be imposed on language, thereby ensuring the inclusion of all potentially relevant studies. Additional studies will be identified by scanning the reference lists of the eligible studies.

Search strategy

The search will be conducted using the following key terms: “Sleep”, “Sleep Initiation and Maintenance Disorders”, “Sleep Wake Disorders”, “Sleep Quality”, “Postoperative Period”, “Delirium”, and “Cohort”. Only observational studies, including cohort, cross-sectional or case-control designs, will be included. The search strategies for each database are listed in S2 File.

Study selection

The records retrieved from the database search will be deduplicated in The Endnote X9 software reference manager. Then, two reviewers (QLH and CHS) will independently review the titles and abstracts according to the eligibility criteria. Full-text articles of the remaining records will be assessed against the eligibility criteria. Any disagreements will be resolved by consensus. The reasons for excluding full-text studies will be recorded and reported.

Data extraction

Two independent reviewers will extract the data from included studies using a Microsoft Excel spreadsheet. Any disagreements in data extraction will be resolved by discussion or consultation with a third reviewer. The following information will be collected: study characteristics (first author, publication year, age, country, number of patients, study design,

type of surgery), sleep quality assessment details (sleep quality assessment tool, the timing of sleep disorder assessment, type of sleep disorder assessed, number of patients with good and poor sleep quality), delirium assessment details (delirium assessment tool, number of patients with and without delirium). In cases of missing or unclear data, the corresponding authors will be contacted for obtain the relevant information. If the number of the case group and the control group are not available by literature or mail, the study will be excluded.

Quality assessment

The Newcastle Ottawa Scale (NOS) assessment scale will be used to evaluate the quality of the included studies[18]. This assessment tool covers three domains: selection, comparability, and outcome or exposure, with score ranges from 0 to 9. High quality researches will be defined as seven or more scores, moderate quality as five to six scores and low quality as less than five. Any disagreements will be resolved by consensus.

Publication bias

Publication bias will be visually evaluated by the funnel plot. Any asymmetry detected in funnel plots will imply the presence of bias. Additionally, Egger's tests will be used to assess the severity of publication bias, with $P<0.05$ considered statistically significant.

Certainty of evidence

Two independent reviewers (QLH and CHS) will assess the meta-analysis results using the Grading of Recommendations, Assessment, Development and Evaluation (GRADE) approach[19]. We will summarize the quality of evidence across five dimensions, including risk of bias, indirectness of evidence, inconsistency in results, imprecision, and potential publication biases. According to GRADE, the quality of evidence can be qualified in one of four levels: high,

moderate, low or very low. Any disagreements will be resolved through discussion or by consulting a third reviewer.

Data synthesis

In cases where two or more studies are available, reporting on the same outcome measures, a qualitative synthesis will be used to summarize the results of all included studies, otherwise narrative synthesis will be taken.

The meta-analysis will be conducted by RevMan 5.3 (Cochrane collaboration, Oxford, UK) and Stata 14(Stata Corp, College Station, TX, USA), considering a two-sided and p value less than 0.05 as statistically significant. The association between sleep disorders and POD will be evaluated by calculating the pooled odds ratios and associated 95% confidence intervals. The degree of heterogeneity was evaluated based on the Cochran's Q statistics and I^2 statistics; a value greater than 50% and $p<0.05$ indicated substantial heterogeneity between studies. Owing to differences in study design and settings, we used the random-effects model[20] to pool all odds ratios. If a study qualifies for inclusion in the systematic review but doesn't supply enough data for the meta-analysis, we'll also perform a systematic review along with a descriptive analysis.

When substantial heterogeneity is detected, we will investigate sources of heterogeneity by using meta-regression analysis or subgroup analysis. Subgroup analysis will be stratified by variables, such as study design (retrospective, prospective), mean population age (greater than 65, less than 65), sleep disorder type (i.e., insomnia, poor sleep quality, obstructive sleep apnoea, and unspecified sleep disorder), timing of sleep disorder (preoperative, postoperative before delirium), study country (China, outside China), and type of surgery (cardiac surgery,

non-cardiac surgery) to determine whether other factors affected the findings. To further explore the sources of heterogeneity, meta-regression analyses will also be conducted, variables such as sample size, publication year, and percentage of female participants will be taken into account.

Additionally, sensitivity analysis will also be used to test whether the pooled results are reliable. Sensitivity analyses will be conducted using the leave-one-out method to evaluate the influence of each study. Finally, the evidence quality will be assessed by the GRADE approach.

Discussion

Many studies have investigated the correlation between sleep disorders exposure and POD outcomes[5, 21]. Sleep disorders are prevalent and common during the surgery, and may increase the risk of POD. The objective of this study is to elucidate the association of sleep disorders and the risk of POD in adult patients. The findings of this review and meta-analysis will provide important data for future studies on intervention or treatment. Also, according to the results of the review and meta-analysis, patients with POD may take into consideration the sleep disorders to make better health decisions.

Key limitations of this review need to be considered. First, the different assessment methods of sleep disorders and the different definitions of POD can lead to a higher risk of publication. Therefore, subgroup analysis and meta-regression should be used to explore the source of heterogeneity. Second, it may be hard to establish causal relationships because most of the studies are expected to be observational. The GRADE tool will serve as a reference in the compilation of the summary table for the included studies. In general, our findings will help to improve the outcomes of patients with POD and provide better guidance for patients undergoing

surgery.

Supporting Information

S1 File. PRISMA-P 2015 Checklist.

(DOCX)

S2 File. Search strategies for included databases.

(DOCX)

Data availability

No datasets were generated or analyzed during the current study. All relevant data from this study will be made available when the study is completed and published.

Funding

The research work was funded by a grant from the National Natural Science Foundation of China (grant no. 81971772). The funders had no role in study design, data collection and analysis, decision to publish, or preparation of the manuscript.

Conflicts of Interest

The authors have declared that no competing interests exist.

Author Contributions

Qianli Huang- Conceptualization, Data curation, Formal analysis, Methodology, Software, Writing – original draft, Writing – review & editing

Changhui Shao- Conceptualization, Data curation, Software, Supervision, Writing – review & editing

Ling Peng- Formal analysis, Methodology, Writing – review & editing

Wei Wei- Formal analysis, Methodology, Writing – review & editing

References

1. Inouye SK, Westendorp RG, Saczynski JS. Delirium in elderly people. Lancet (London, England). 2014;383(9920):911-22.
2. Yan E, Veitch M, Saripella A, Alhamdah Y, Butris N, Tang-Wai DF, et al. Association between postoperative delirium and adverse outcomes in older surgical patients: A systematic review and meta-analysis. Journal of clinical anesthesia. 2023;90:111221.
3. Huang H, Li H, Zhang X, Shi G, Xu M, Ru X, et al. Association of postoperative delirium with cognitive outcomes: A meta-analysis. Journal of clinical anesthesia. 2021;75:110496.
4. Bai J, Liang Y, Zhang P, Liang X, He J, Wang J, et al. Association between postoperative delirium and mortality in elderly patients undergoing hip fractures surgery: a meta-analysis. Osteoporosis international : a journal established as result of cooperation between the European Foundation for Osteoporosis and the National Osteoporosis Foundation of the USA. 2020;31(2):317-26.
5. Butris N, Tang E, Pivetta B, He D, Saripella A, Yan E, et al. The prevalence and risk factors of sleep disturbances in surgical patients: A systematic review and meta-analysis. Sleep medicine reviews. 2023;69:101786.
6. Ida M, Onodera H, Yamauchi M, Kawaguchi M. Preoperative sleep disruption and postoperative functional disability in lung surgery patients: a prospective observational study. Journal of anesthesia. 2019;33(4):501-8.
7. Rhon DI, Snodgrass SJ, Cleland JA, Cook CE. Comorbid Insomnia and Sleep Apnea are Associated with Greater Downstream Health Care Utilization and Chronic Opioid Use after Arthroscopic Hip Surgery. Pain physician. 2019;22(4):E351-e60.
8. Wang Y, Jiang Y, Chen T, Xia Q, Wang X, Lv Q, et al. Prediction of risk factors of sleep disturbance in patients undergoing total hip arthroplasty. Sleep and biological rhythms. 2024;22(1):85-91.
9. Lam EWK, Chung F, Wong J. Sleep-Disordered Breathing, Postoperative Delirium, and Cognitive Impairment. Anesthesia and analgesia. 2017;124(5):1626-35.
10. Kjølhede P, Langström P, Nilsson P, Wodlin NB, Nilsson L. The impact of quality of sleep on recovery from fast-track abdominal hysterectomy. Journal of clinical sleep medicine : JCSM : official publication of the American Academy of Sleep Medicine. 2012;8(4):395-402.
11. Niklasson A, Finan PH, Smith MT, Forsberg A, Dietz N, Kander T, et al. The relationship between preoperative sleep disturbance and acute postoperative pain control: A systematic review and meta-analysis. Sleep medicine reviews. 2024;79:102014.
12. Fadayomi AB, Ibala R, Bilotta F, Westover MB, Akeju O. A Systematic Review and Meta-Analysis Examining the Impact of Sleep Disturbance on Postoperative Delirium. Critical care medicine. 2018;46(12):e1204-e12.
13. Wang H, Zhang L, Zhang Z, Li Y, Luo Q, Yuan S, et al. Perioperative Sleep Disturbances and Postoperative Delirium in Adult Patients: A Systematic Review and Meta-Analysis of Clinical Trials. Frontiers in psychiatry. 2020;11:570362.
14. He E, Dong Y, Jia H, Yu L. Relationship of sleep disturbance and postoperative delirium: a systematic review and meta-analysis. Gland surgery. 2022;11(7):1192-203.
15. Huang Z, Huang C, Deng Y, Lu H, Shi L. Association Between Preoperative Long-Term Poor Sleep Quality and Postoperative Delirium in Elderly Patients Undergoing Cardiac Surgery:

A Multi-Center Observational Study. The American surgeon. 2024;90(6):1260-7.
16. Ou-Yang CL, Ma LB, Wu XD, Ma YL, Liu YH, Tong L, et al. Association of sleep quality on the night of operative day with postoperative delirium in elderly patients: A prospective cohort study. European Journal of Anaesthesiology. 2024;41(3):226-33.
17. Page MJ, McKenzie JE, Bossuyt PM, Boutron I, Hoffmann TC, Mulrow CD, et al. The PRISMA 2020 statement: an updated guideline for reporting systematic reviews. BMJ (Clinical research ed). 2021;372:n71.
18. Stang A. Critical evaluation of the Newcastle-Ottawa scale for the assessment of the quality of nonrandomized studies in meta-analyses. European journal of epidemiology. 2010;25(9):603-5.
19. Guyatt GH, Oxman AD, Vist GE, Kunz R, Falck-Ytter Y, Alonso-Coello P, et al. GRADE: an emerging consensus on rating quality of evidence and strength of recommendations. BMJ (Clinical research ed). 2008;336(7650):924-6.
20. DerSimonian R, Laird N. Meta-analysis in clinical trials. Controlled clinical trials. 1986;7(3):177-88.
21. Lin D, Huang X, Sun Y, Wei C, Wu A. Perioperative Sleep Disorder: A Review. Frontiers in medicine. 2021;8:640416.

S1

Click here to access/download
Supporting Information
S1 File.docx

S2

Click here to access/download
Supporting Information
S2 File.docx
