# Supplementary material for: The relationship between sleep disorders and postoperative delirium in adult patients: Protocol for an updated systematic review and meta-analysis
Source: PLoS One. 2025 Dec 2;20(12):e0337598. doi: 10.1371/journal.pone.0337598 (PMC12671798; doi:10.1371/journal.pone.0337598)
Supplement: Attachment — Submitted filename: PONE-D-25-29095_R1_fc.pdf [file pone.0337598.s005.pdf]

PLOS ONE

The relationship between sleep disorders and postoperative delirium in adult patients: protocol for an updated systematic review and meta-analysis

--Manuscript Draft--

**Manuscript Number:**	PONE-D-25-29095R1
**Article Type:**	Study Protocol
**Full Title:**	The relationship between sleep disorders and postoperative delirium in adult patients: protocol for an updated systematic review and meta-analysis
**Short Title:**	The relationship between sleep disorders and postoperative delirium in adult patients
**Corresponding Author:**	Wei Wei<br>West China Hospital of Sichuan University<br>Chengdu, CHINA
**Keywords:**	Sleep disorders; Postoperative Delirium; adult; Meta-analysis
**Abstract:**	Introduction: Postoperative delirium (POD) is a common complication after surgery. The association between sleep disorders and the risk of POD has been widely studied. Sleep disorders have emerged as a potential risk factor for POD, but recent studies provide conflicting evidence regarding the existence and the extent of the association. The aim of this systematic review and meta-analysis will be to estimate the association between sleep disorders and the risk of POD in adult patients.<br>Methods and analysis：A systematic review will be conducted to estimate the association between sleep disorders and the risk of POD in adult patients. This systematic review protocol follows the Preferred Reporting Items for Systematic Review and Meta-Analysis Protocols (PRISMA-P) statement. Literature searches will be carried out in the Pubmed, Embase, CINAHL, and PsycINFO databases from inception until August 2025 without language restrictions. Only observational studies that investigated the association between sleep disorders and POD will be included. Two independent reviewers will be responsible for the selection of studies, extraction of information and risk of bias assessment of the articles. A random effects model will be used to calculate the pooled risk estimates for the outcome. Subgroup analysis will be used to explore potential sources of heterogeneity. Publication bias will be estimated by funnel plots and Egger's test. Sensitivity analysis will also be performed to explore the stability of the overall effect size. Also, evidence quality will be assessed. All data will be analysed using Review Manager (V.5.3) and Stata (V.14.0) statistical software.<br>Discussion: The proposed systematic review and meta-analysis will highlight the association of sleep disorders and the risk of POD in adult patients. The findings of this review and meta-analysis will help clinicians consider the sleep disorders to make better health decisions.<br>Trial registration: Prospero registration number: CRD42024604118
**Order of Authors:**	Qianli Huang
	Changhui Shao
	Ling Peng
	Wei Wei
**Response to Reviewers:**	Dear Editor and Reviewers<br>Thank you very much for giving us the opportunity to revise our manuscript (PONE-D-25-29095). We sincerely appreciate the valuable comments and suggestions provided by the reviewers, which have significantly helped us to improve the quality of our manuscript. We have addressed each of the reviewers' comments in detail below.<br>Journal requirements:<br>1.Please ensure that your manuscript meets PLOS ONE's style requirements, including those for file naming.<br>Reply: We have carefully checked throughout the manuscript.<br>2. Thank you for stating the following financial disclosure:<br>[The research work was funded by a grant from the National Natural Science Foundation of China (grant no. 81971772).].<br>Please state what role the funders took in the study. If the funders had no role, please

Powered by Editorial Manager® and ProduXion Manager® from Aries Systems Corporation

state: "The funders had no role in study design, data collection and analysis, decision to publish, or preparation of the manuscript."
If this statement is not correct you must amend it as needed.
Please include this amended Role of Funder statement in your cover letter; we will change the online submission form on your behalf.
Reply: The funders had no role in study design, data collection and analysis, decision to publish, or preparation of the manuscript.
3. Please remove any funding-related text from the manuscript and let us know how you would like to update your Funding Statement. Currently, your Funding Statement reads as follows:
[The research work was funded by a grant from the National Natural Science Foundation of China (grant no. 81971772).]
Please include your amended statements within your cover letter; we will change the online submission form on your behalf.
Reply: we have removed any funding-related text from the manuscript and included our amended statements within cover letter.
Comments to the Author
Reviewer #1:
1. Pg 3: Financial disclosure statement missing
Reply: Thank you for your suggestion. We have added the financial disclosure statement.
2. Pg 6 ln 120-121: ? does this include pre, peri and post op period? or just the perioperative period? Please clarify as earlier on you are presenting data from all periods?
Reply: We have modified the description of the perioperative sleep disorders. The timing of sleep disorders includes either the pre- or post-operative period prior to delirium.
3. Pg 6 ln 123: ?This is not an outcome. You need to define what outcomes you are specifically measuring - e.g. incidence of POD
Reply: Thank you very much for your valuable comments, we have revised the outcomes.
4. Pg 6 ln 124-125: If you are looking for an association between the exposure and outcome, then why are you including cross sectional studies? Also, you discussed conflicts within literature between observational studies and RCT, yet you are excluding RCT - please justify your reasoning behind this.
Reply: Thank you very much for pointing out this issue. Given that cross-sectional studies cannot determine the association between the exposure and outcome, we have excluded the cross-sectional studies. In recent years, several observational studies have explored the association between sleep disorders and POD; however, few newly published RCTs have addressed this topic, so this review could not provide new insights into the association between sleep disorders and POD based on RCTs.
5. Pg 6 ln 128-19: the use of insomnia symptoms or poor sleep quality are subjective measures, and thus if you are going to include them you need to clearly define what is meant by these. Also, how is poor sleep quality being identified? Using well defined criteria/diagnoses would provide a more objective and standard/consistent measure for sleep disorders.
Reply: Thank you very much for your valuable comments. We have provided a more objective and standard/consistent measure for sleep disorders.
6. Pg 6 ln 132: Also RCT according to your earlier criteria.
Reply: Thank you for your suggestions. We have added this information in the exclusion criteria.
7. Pg 7 ln" 134: I am curious why Embase is being used, given its primary focus is on pharmaceuticals/drugs?
What about alternative databases, like CINAHL, especially as you indicate you are only including observational studies?
Reply: Thank you for your suggestions. We have added the CINAHL database for a comprehensive search strategy according to the suggested idea.
8. Pg 16 ln 194-199: what about the pre-existence of cognitive impairment/dementia, which is an important influencing factor, that can also impact on the diagnosis of POD?
Reply: Thank you very much for this good question. We have revised the participants of inclusion criteria.
Reviewer #2:
1. I have some concerns about the methodology you propose to study this topic. You indicate that your search will be limited to observational studies; however, you plan to

	search Cochrane Library, which indexes systematic reviews (CDSR) and randomized controlled trials (Central). This seems contradictory. Similarly, you justify your study by noting that since the publication of prior reviews there have been additional studies published which may contribute to further understanding; however, both of the additional studies cited appear to concern preoperative sleep disturbances, not postoperative.<br>Reply: Thank you very much for this good question. We have deleted the Cochrane Library. We have added this information in the manuscript as “However, since the publication of the previous meta-analysis, there have been additional studies published with large-scale investigations[15, 16]. Considering the importance of the topic, the aim of this study is to update the current evidence on the relationship between sleep disorders and the risk of POD.” The cited study, "Association of sleep quality on the night of the operative day with postoperative delirium in elderly patients: A prospective cohort study," explored the association between postoperative sleep disturbances and POD.<br>2. Your search strategy also has issues that limit the methodological quality of the proposed review. You are missing potential synonyms such as emergence agitation and post surgical (with space). There are also unexplained differences in free-text search terms between different databases. Suggest also considering a search of PsycINFO, given the topic area.<br>Reply: Thank you for pointing this out. We have added keywords such as "emergence agitation" and "post-surgical," as well as the PsycINFO database.<br>In summary, we have incorporated all the reviewers’ suggestions to the best of our ability, and we believe the revised manuscript has been significantly improved as a result. A detailed list of changes is highlighted in the revised manuscript.
**Additional Information:**	
**Question**	**Response**
**Financial Disclosure**<br><br>Enter a financial disclosure statement that describes the sources of funding for the work included in this submission. Review the submission guidelines for detailed requirements. View published research articles from *PLOS ONE* for specific examples.<br><br>This statement is required for submission and **will appear in the published article** if the submission is accepted. Please make sure it is accurate.<br><br>**Funded studies**<br>Enter a statement with the following details:<br>• Initials of the authors who received each award<br>• Grant numbers awarded to each author<br>• The full name of each funder<br>• URL of each funder website<br>• Did the sponsors or funders play any role in the study design, data collection and analysis, decision to publish, or preparation of the manuscript?	Yes

Did you receive funding for this work?	
Please add funding details.<br>as follow-up to "**Financial Disclosure**<br><br>Enter a financial disclosure statement that describes the sources of funding for the work included in this submission. Review the submission guidelines for detailed requirements. View published research articles from *PLOS ONE* for specific examples.<br><br>This statement is required for submission and **will appear in the published article** if the submission is accepted. Please make sure it is accurate.<br><br>**Funded studies**<br>Enter a statement with the following details:<br>• Initials of the authors who received each award<br>• Grant numbers awarded to each author<br>• The full name of each funder<br>• URL of each funder website<br>• Did the sponsors or funders play any role in the study design, data collection and analysis, decision to publish, or preparation of the manuscript?<br><br>Did you receive funding for this work?"	The research work was funded by a grant from the National Natural Science Foundation of China (grant no. 81971772). The funders had no role in study design, data collection and analysis, decision to publish, or preparation of the manuscript
Please select the country of your main research funder (please select carefully as in some cases this is used in fee calculation).<br>as follow-up to "**Financial Disclosure**<br><br>Enter a financial disclosure statement that describes the sources of funding for the work included in this submission. Review the submission guidelines for detailed requirements. View published research articles from *PLOS ONE* for specific examples.<br><br>This statement is required for submission and **will appear in the published article** if	CHINA - CN

the submission is accepted. Please make sure it is accurate.<br><br>**Funded studies**<br>Enter a statement with the following details:<br>• Initials of the authors who received each award<br>• Grant numbers awarded to each author<br>• The full name of each funder<br>• URL of each funder website<br>• Did the sponsors or funders play any role in the study design, data collection and analysis, decision to publish, or preparation of the manuscript?<br><br>Did you receive funding for this work?"	
**Competing Interests**<br><br>Use the instructions below to enter a competing interest statement for this submission. On behalf of all authors, disclose any competing interests that could be perceived to bias this work—acknowledging all financial support and any other relevant financial or non-financial competing interests.<br><br>This statement is required for submission and **will appear in the published article** if the submission is accepted. Please make sure it is accurate and that any funding sources listed in your Funding Information later in the submission form are also declared in your Financial Disclosure statement.<br><br>View published research articles from *PLOS ONE* for specific examples.	The authors have declared that no competing interests exist.

**NO authors have competing interests**<br><br>Enter: *The authors have declared that no competing interests exist.*<br><br>**Authors with competing interests**<br><br>Enter competing interest details beginning with this statement:<br><br>*I have read the journal's policy and the authors of this manuscript have the following competing interests: [insert competing interests here]*<br><br>* typeset	
**Ethics Statement**<br><br>Enter an ethics statement for this submission. This statement is required if the study involved:<br><br>• Human participants<br>• Human specimens or tissue<br>• Vertebrate animals or cephalopods<br>• Vertebrate embryos or tissues<br>• Field research<br><br>Write "N/A" if the submission does not require an ethics statement.<br><br>General guidance is provided below. Consult the submission guidelines for detailed instructions. **Make sure that all information entered here is included in the Methods section of the manuscript.**	N/A

Powered by Editorial Manager® and ProduXion Manager® from Aries Systems Corporation

**Format for specific study types**<br><br>**Human Subject Research (involving human participants and/or tissue)**<br>• Give the name of the institutional review board or ethics committee that approved the study<br>• Include the approval number and/or a statement indicating approval of this research<br>• Indicate the form of consent obtained (written/oral) or the reason that consent was not obtained (e.g. the data were analyzed anonymously)<br><br>**Animal Research (involving vertebrate animals, embryos or tissues)**<br>• Provide the name of the Institutional Animal Care and Use Committee (IACUC) or other relevant ethics board that reviewed the study protocol, and indicate whether they approved this research or granted a formal waiver of ethical approval<br>• Include an approval number if one was obtained<br>• If the study involved *non-human primates*, add *additional details* about animal welfare and steps taken to ameliorate suffering<br>• If anesthesia, euthanasia, or any kind of animal sacrifice is part of the study, include briefly which substances and/or methods were applied<br><br>**Field Research**<br><br>Include the following details if this study involves the collection of plant, animal, or other materials from a natural setting:<br>• Field permit number<br>• Name of the institution or relevant body that granted permission	
**Data Availability**<br><br>Authors are required to make the data underlying their research findings fully available, without restriction.<br><br>If the manuscript reports pilot or preliminary results, the data underlying those results must be made available at the time of publication. A Data Availability	N/A - No results are reported

Statement describing where the data can be found is required at submission. Your answers to this question constitute the Data Availability Statement and will be published in the article, if accepted. When legal or ethical requirements prohibit public data sharing, authors must indicate how researchers may obtain access to the data. See the PLOS Data Policy and FAQ for detailed information.<br><br>**Important:** Stating 'data available on request from the author' is not sufficient. If your data are only available upon request, select 'No' for the first question and explain your situation in the text box.<br><br>For protocols without pilot or preliminary data, authors are strongly encouraged to state how they plan to share research data from their study when it is completed or published.<br><br>If your study includes pilot data, will they be made fully available without restriction?	
**Describe where the data may be found in full sentences. If you are copying our sample text, replace any instances of XXX with the appropriate details.**<br><br>• If **no pilot data are reported** in the manuscript, please enter the following: *No datasets were generated or analysed during the current study. All relevant data from this study will be made available upon study completion.*<br>• If the manuscript reports pilot data **held in a public repository**, include URLs, accession numbers or DOIs. If this information will only be available after acceptance, indicate this by ticking the box below. For example: *All XXX files are available from the XXX database (accession number(s) XXX, XXX.).*<br>• If the manuscript reports pilot data contained **within the manuscript and/or**	All relevant data are within the manuscript and its Supporting Information files.

**Supporting Information files**, enter the following:<br>*All relevant data are within the manuscript and its Supporting Information files.*<br>• If neither of these applies but you are able to provide **details of access elsewhere**, with or without limitations, please do so. For example:<br><br>*Data cannot be shared publicly because of [XXX]. Data are available from the XXX Institutional Data Access / Ethics Committee (contact via XXX) for researchers who meet the criteria for access to confidential data.*<br><br>*The data underlying the results presented in the study are available from (include the name of the third party and contact information or URL).*<br>• This text is appropriate if the data are owned by a third party and authors do not have permission to share the data.<br><br>For study protocols with data management or sharing plans, authors are encouraged to briefly describe in their Data Availability Statement how data they generate will be made accessible when the study is completed. For example:<br>• Deidentified research data will be made publicly available when the study is completed and published.<br><br>* typeset	
Additional data availability information:	

Powered by Editorial Manager® and ProduXion Manager® from Aries Systems Corporation

Manuscript Click here to access/download;Manuscript;Manuscript.docx

The relationship between sleep disorders and postoperative delirium in adult patients: protocol for an updated systematic review and meta-analysis

Qianli Huang[1 2#], Changhui Shao[3#], Ling Peng[1], Wei Wei[1*]

[1]Department of Anesthesiology, West China Hospital, Sichuan University, Chengdu, Sichuan, China.

[2]Department of Anesthesiology, Chengdu Women's and Children's Central Hospital, School of Medicine, University of Electronic Science and Technology of China, Chengdu, Sichuan, China.

[3]Department of Anesthesiology, Chengdu integrated TCM & Western Medicine Hospital, Chengdu, Sichuan, China.

*Corresponding author

[#] These authors contributed equally to this work

Emails of authors: 1172430737@qq.com (HQL); 814660661@qq.com (CHS); penglingjq@163.com (LP); weiw@scu.edu.cn (WW).

Abstract

Introduction: Postoperative delirium (POD) is a common complication after surgery. The association between sleep disorders and the risk of POD has been widely studied. Sleep disorders have emerged as a potential risk factor for POD, but recent studies provide conflicting evidence regarding the existence and the extent of the association. The aim of this systematic review and meta-analysis will be to estimate the association between sleep disorders and the risk of POD in adult patients.

Methods and analysis: A systematic review will be conducted to estimate the association between sleep disorders and the risk of POD in adult patients. This systematic review protocol follows the Preferred Reporting Items for Systematic Review and Meta-Analysis Protocols (PRISMA-P) statement. Literature searches will be carried out in the Pubmed, Embase, CINAHL, and PsycINFO databases from inception until August 2025 without language restrictions. Only observational studies that investigated the association between sleep disorders and POD will be included. Two independent reviewers will be responsible for the selection of studies, extraction of information and risk of bias assessment of the articles. A random effects model will be used to calculate the pooled risk estimates for the outcome. Subgroup analysis will be used to explore potential sources of heterogeneity. Publication bias will be estimated by funnel plots and Egger's test. Sensitivity analysis will also be performed to explore the stability of the overall effect size. Also, evidence quality will be assessed. All data will be analysed using Review Manager (V.5.3) and Stata (V.14.0) statistical software.

Discussion: The proposed systematic review and meta-analysis will highlight the association of sleep disorders and the risk of POD in adult patients. The findings of this review

and meta-analysis will help clinicians consider the sleep disorders to make better health decisions.

Trial registration: Prospero registration number: CRD42024604118

Introduction

Postoperative delirium (POD) a is common and serious postoperative complication, affecting up to 50% of patients in some cohorts[1]. It is associated with a range of adverse outcomes, such as prolonged hospital stays, elevated healthcare costs, cognitive impairment, and higher risk of mortality and morbidity[2-4]. Given the significant adverse outcomes associated with POD, it is crucial for clinicians to identify risk factors and quantify their impact on POD.

Sleep is a complex physiological process that is essential for overall health and well-being. Sleep disorders are highly prevalent during the perioperative period, including conditions such as insomnia, sleep-disordered breathing, and circadian rhythm disruption. Recent studies have shown that 8.8%-79.1% of patients complained about poor sleep quality in the preoperative period[5-7]. Additionally, postoperative sleep disorders are highly prevalent in the first 2 weeks after surgery[8]. In the context of surgical patients, perioperative sleep disturbances may be associated with adverse prognostic outcomes, such as higher incidence of delirium and cognitive impairment, increased postoperative pain intensity, delayed postoperative recovery, increased hospital length of stay, and poor quality of life[9-11].

Extensive research has investigated the relationship between sleep disorders and the risk of POD. An earlier meta-analysis concluded that pre-operative sleep disturbances are likely associated with POD[12]. However, whether postoperative sleep disturbances serve as a risk factor for POD is still unknown. Initially, a meta-analysis conducted by Wang et al. had investigated association between perioperative sleep disturbance and the risk of POD[13]. Results showed that perioperative sleep disturbances were associated with an increased risk of POD in observational trials, yet no significant association was detected in randomized

controlled trials. Meanwhile, this study was limited by high heterogeneity and a small sample size, which may have affected the generalizability and statistical power of the findings. A recent meta-analysis incorporated 16 observational studies which concluded that sleep disturbance may increase the risk of POD[14]. This analysis offered valuable insights but was limited by relatively small sample sizes and the inclusion of only English-language literature.

However, since the publication of the previous meta-analysis, there have been additional studies published with large-scale investigations[15, 16]. Considering the importance of the topic, the aim of this study is to update the current evidence on the relationship between sleep disorders and the risk of POD.

Methods

Protocol and registration

This systematic review and meta-analysis was performed in accordance with the Preferred Reporting Items for Systematic Reviews and Meta-Analyses (PRISMA) guidelines[17] and the study protocol was registered in the International Prospective Register of Systematic Reviews (PROSPERO) (registration number: CRD42024604118). The PRISMA-P-checklist is shown in S1 File.

Ethics and dissemination

As this systematic review is summary and analysis of existing literature, there is no requirement for ethics approval. The results of the study will be published in a peer-reviewed journal.

Patient and public involvement

Patients and/or the public were not involved in the design, or conduct, or reporting, or dissemination plans of this research.

Objectives and outcomes

The sole objective of this is to determine whether sleep disorders are associated with an increased risk of POD in adult patients after surgery. The study aims to answer the research questions based on PECOS (Population, Exposure, Compares, Outcomes and Study design) criteria:

What is the association between sleep disorders and the risk of POD in adult patients after surgery?

PECOS eligibility criteria

- Population: adult patients aged 18 years and older undergoing surgery without pre-existing delirium/neurocognitive disorders before admission.
- Exposure: studies reporting on sleep disorders as exposure variables, defined as those occurring in either the pre- or post-operative period prior to delirium.
- Comparator: the population without exposure to sleep disorders served as a comparator
- Outcome: the occurrence of POD measured by a validated scale, such as the 5th edition of Diagnostic and Statistical Manual of Mental Disorders (DSM-5), the Confusion Assessment Method (CAM), or Confusion Assessment Method for the Intensive Care Unit (CAM-ICU).
- Study design: We will only include observational studies (retrospective or prospective cohort studies, or case-control studies).

The inclusion criteria are as follows:

1. Studies investigating the association of sleep disorders and the risk of POD.
2. Included participants diagnosed with sleep disorders and no sleep disorders. The included

studies should report the subjectively or objectively diagnostic criteria for sleep disorders. Subjectively measured sleep disorders will be considered self-reported or obtained by any questionnaires (e.g., Pittsburgh Sleep Quality Index). Data from polysomnography (PSG) or actigraphy will be considered for objective measures of sleep disorders.

The exclusion criteria are as follows:

1. Studies without measurement of sleep disorders or POD.
2. Randomized controlled trials (RCTs), cross-sectional studies, review, meta-analyses, letter, editorial, protocol, case reports, or animal experiments.

Information sources

We will conduct searches in the following databases, including PubMed, Embase, CINAHL, and PsycINFO, for studies published before August 2025, that reported on the association between sleep disorders and postoperative delirium in adult patients. No restrictions will be imposed on language, thereby ensuring the inclusion of all potentially relevant studies. Additional studies will be identified by scanning the reference lists of the eligible studies.

Search strategy

The employed search keywords will include variations related to sleep, such as: ‘sleep’, ‘sleep initiation and maintenance disorders’, ‘sleep wake disorders’, and ‘sleep quality’, combined with terms associated with POD, including ‘postoperative delirium’, ‘delirium’, ‘emergence agitation’, ‘confusion’, ‘postoperative’, ‘surgery’ and ‘anesthesia’. Only observational studies, including cohort or case-control designs, will be included. The draft of the search strategy for each database is listed in S2 File.

Study selection

The records retrieved from the database search will be deduplicated in The Endnote X9 software reference manager. Then, two reviewers (QLH and CHS) will independently review the titles and abstracts according to the eligibility criteria. Full-text articles of the remaining records will be assessed against the eligibility criteria. Any disagreements will be resolved by consensus. The reasons for excluding full-text studies will be recorded and reported.

Data extraction

Two independent reviewers will extract the data from included studies using a Microsoft Excel spreadsheet. Any disagreements in data extraction will be resolved by discussion or consultation with a third reviewer. The following information will be collected: study characteristics (first author, publication year, age, country, number of patients, study design, type of surgery), sleep quality assessment details (sleep quality assessment tool, the timing of sleep disorder assessment, type of sleep disorder assessed, number of patients with good and poor sleep quality), delirium assessment details (delirium assessment tool, number of patients with and without delirium). In cases of missing or unclear data, the corresponding authors will be contacted for obtain the relevant information. If the number of the case group and the control group are not available by literature or mail, the study will be excluded.

Quality assessment

The Newcastle Ottawa Scale (NOS) assessment scale will be used to evaluate the quality of the included studies[18]. This assessment tool covers three domains: selection, comparability, and outcome or exposure, with score ranges from 0 to 9. High quality researches will be defined as seven or more scores, moderate quality as five to six scores and low quality as less than five. Any disagreements will be resolved by consensus.

Publication bias

Publication bias will be visually evaluated by the funnel plot. Any asymmetry detected in funnel plots will imply the presence of bias. Additionally, Egger's tests will be used to assess the severity of publication bias, with $P<0.05$ considered statistically significant.

Certainty of evidence

Two independent reviewers (QLH and CHS) will assess the meta-analysis results using the Grading of Recommendations, Assessment, Development and Evaluation (GRADE) approach[19]. We will summarize the quality of evidence across five dimensions, including risk of bias, indirectness of evidence, inconsistency in results, imprecision, and potential publication biases. According to GRADE, the quality of evidence can be qualified in one of four levels: high, moderate, low or very low. Any disagreements will be resolved through discussion or by consulting a third reviewer.

Data synthesis

In cases where two or more studies are available, reporting on the same outcome measures, a qualitative synthesis will be used to summarize the results of all included studies, otherwise narrative synthesis will be taken.

The meta-analysis will be conducted by RevMan 5.3 (Cochrane collaboration, Oxford, UK) and Stata 14(Stata Corp, College Station, TX, USA), considering a two-sided and p value less than 0.05 as statistically significant. The association between sleep disorders and POD will be evaluated by calculating the pooled odds ratios and associated 95% confidence intervals. The degree of heterogeneity was evaluated based on the Cochran’s Q statistics and I^2 statistics; a value greater than 50% and $p<0.05$ indicated substantial heterogeneity between studies. Owing

to differences in study design and settings, we used the random-effects model[20] to pool all odds ratios. If a study qualifies for inclusion in the systematic review but doesn't supply enough data for the meta-analysis, we'll also perform a systematic review along with a descriptive analysis.

When substantial heterogeneity is detected, we will investigate sources of heterogeneity by using meta-regression analysis or subgroup analysis. Subgroup analysis will be stratified by variables, such as study design (retrospective, prospective), mean population age (greater than 65, less than 65), sleep disorder type (i.e., insomnia, poor sleep quality, obstructive sleep apnoea, and unspecified sleep disorder), timing of sleep disorder (preoperative, postoperative before delirium), study country (China, outside China), and type of surgery (cardiac surgery, non-cardiac surgery) to determine whether other factors affected the findings. To further explore the sources of heterogeneity, meta-regression analyses will also be conducted, variables such as sample size, publication year, and percentage of female participants will be taken into account.

Additionally, sensitivity analysis will also be used to test whether the pooled results are reliable. Sensitivity analyses will be conducted using the leave-one-out method to evaluate the influence of each study. Finally, the evidence quality will be assessed by the GRADE approach.

Discussion

Many studies have investigated the correlation between sleep disorders exposure and POD outcomes[5, 21]. Sleep disorders are prevalent and common during the surgery, and may increase the risk of POD. The objective of this study is to elucidate the association of sleep disorders and the risk of POD in adult patients. The findings of this review and meta-analysis

will provide important data for future studies on intervention or treatment. Also, according to the results of the review and meta-analysis, patients with POD may take into consideration the sleep disorders to make better health decisions.

Key limitations of this review need to be considered. First, the different assessment methods of sleep disorders and the different definitions of POD can lead to a higher risk of publication. Therefore, subgroup analysis and meta-regression should be used to explore the source of heterogeneity. Second, it may be hard to establish causal relationships because most of the studies are expected to be observational. The GRADE tool will serve as a reference in the compilation of the summary table for the included studies. In general, our findings will help to improve the outcomes of patients with POD and provide better guidance for patients undergoing surgery.

Supporting Information

S1 File. PRISMA-P 2015 Checklist.

(DOCX)

S2 File. Search strategies for included databases.

(DOCX)

Data availability

No datasets were generated or analyzed during the current study. All relevant data from this study will be made available when the study is completed and published.

Conflicts of Interest

The authors have declared that no competing interests exist.

Author Contributions

Qianli Huang- Conceptualization, Data curation, Formal analysis, Methodology, Software, Writing – original draft, Writing – review & editing

Changhui Shao- Conceptualization, Data curation, Software, Supervision, Writing – review & editing

Ling Peng- Formal analysis, Methodology, Writing – review & editing

Wei Wei- Formal analysis, Methodology, Writing – review & editing

References

1. Inouye SK, Westendorp RG, Saczynski JS. Delirium in elderly people. Lancet (London, England). 2014;383(9920):911-22.

2. Yan E, Veitch M, Saripella A, Alhamdah Y, Butris N, Tang-Wai DF, et al. Association between postoperative delirium and adverse outcomes in older surgical patients: A systematic review and meta-analysis. Journal of clinical anesthesia. 2023;90:111221.

3. Huang H, Li H, Zhang X, Shi G, Xu M, Ru X, et al. Association of postoperative delirium with cognitive outcomes: A meta-analysis. Journal of clinical anesthesia. 2021;75:110496.

4. Bai J, Liang Y, Zhang P, Liang X, He J, Wang J, et al. Association between postoperative delirium and mortality in elderly patients undergoing hip fractures surgery: a meta-analysis. Osteoporosis international : a journal established as result of cooperation between the European Foundation for Osteoporosis and the National Osteoporosis Foundation of the USA. 2020;31(2):317-26.

5. Butris N, Tang E, Pivetta B, He D, Saripella A, Yan E, et al. The prevalence and risk factors of sleep disturbances in surgical patients: A systematic review and meta-analysis. Sleep medicine reviews. 2023;69:101786.

6. Ida M, Onodera H, Yamauchi M, Kawaguchi M. Preoperative sleep disruption and postoperative functional disability in lung surgery patients: a prospective observational study. Journal of anesthesia. 2019;33(4):501-8.

7. Rhon DI, Snodgrass SJ, Cleland JA, Cook CE. Comorbid Insomnia and Sleep Apnea are Associated with Greater Downstream Health Care Utilization and Chronic Opioid Use after Arthroscopic Hip Surgery. Pain physician. 2019;22(4):E351-e60.

8. Wang Y, Jiang Y, Chen T, Xia Q, Wang X, Lv Q, et al. Prediction of risk factors of sleep disturbance in patients undergoing total hip arthroplasty. Sleep and biological rhythms. 2024;22(1):85-91.

9. Lam EWK, Chung F, Wong J. Sleep-Disordered Breathing, Postoperative Delirium, and Cognitive Impairment. Anesthesia and analgesia. 2017;124(5):1626-35.

10. Kjølhede P, Langström P, Nilsson P, Wodlin NB, Nilsson L. The impact of quality of sleep on recovery from fast-track abdominal hysterectomy. Journal of clinical sleep medicine : JCSM : official publication of the American Academy of Sleep Medicine. 2012;8(4):395-402.

11. Niklasson A, Finan PH, Smith MT, Forsberg A, Dietz N, Kander T, et al. The relationship between preoperative sleep disturbance and acute postoperative pain control: A systematic review and meta-analysis. Sleep medicine reviews. 2024;79:102014.

12. Fadayomi AB, Ibala R, Bilotta F, Westover MB, Akeju O. A Systematic Review and Meta-Analysis Examining the Impact of Sleep Disturbance on Postoperative Delirium. Critical care medicine. 2018;46(12):e1204-e12.

13. Wang H, Zhang L, Zhang Z, Li Y, Luo Q, Yuan S, et al. Perioperative Sleep Disturbances and Postoperative Delirium in Adult Patients: A Systematic Review and Meta-Analysis of

Clinical Trials. Frontiers in psychiatry. 2020;11:570362.

14. He E, Dong Y, Jia H, Yu L. Relationship of sleep disturbance and postoperative delirium: a systematic review and meta-analysis. Gland surgery. 2022;11(7):1192-203.

15. Huang Z, Huang C, Deng Y, Lu H, Shi L. Association Between Preoperative Long-Term Poor Sleep Quality and Postoperative Delirium in Elderly Patients Undergoing Cardiac Surgery: A Multi-Center Observational Study. The American surgeon. 2024;90(6):1260-7.

16. Ou-Yang CL, Ma LB, Wu XD, Ma YL, Liu YH, Tong L, et al. Association of sleep quality on the night of operative day with postoperative delirium in elderly patients: A prospective cohort study. European Journal of Anaesthesiology. 2024;41(3):226-33.

17. Page MJ, McKenzie JE, Bossuyt PM, Boutron I, Hoffmann TC, Mulrow CD, et al. The PRISMA 2020 statement: an updated guideline for reporting systematic reviews. BMJ (Clinical research ed). 2021;372:n71.

18. Stang A. Critical evaluation of the Newcastle-Ottawa scale for the assessment of the quality of nonrandomized studies in meta-analyses. European journal of epidemiology. 2010;25(9):603-5.

19. Guyatt GH, Oxman AD, Vist GE, Kunz R, Falck-Ytter Y, Alonso-Coello P, et al. GRADE: an emerging consensus on rating quality of evidence and strength of recommendations. BMJ (Clinical research ed). 2008;336(7650):924-6.

20. DerSimonian R, Laird N. Meta-analysis in clinical trials. Controlled clinical trials. 1986;7(3):177-88.

21. Lin D, Huang X, Sun Y, Wei C, Wu A. Perioperative Sleep Disorder: A Review. Frontiers in medicine. 2021;8:640416.

S1 File

Click here to access/download
Supporting Information
S1 File.docx

S2 File

Click here to access/download
Supporting Information
S2 File.docx

The relationship between sleep disorders and postoperative delirium in adult patients: protocol for an updated systematic review and meta-analysis

Qianli Huang[1 2#], Changhui Shao[3#], Ling Peng[1], Wei Wei[1*]

[1]Department of Anesthesiology, West China Hospital, Sichuan University, Chengdu, Sichuan, China.

[2]Department of Anesthesiology, Chengdu Women's and Children's Central Hospital, School of Medicine, University of Electronic Science and Technology of China, Chengdu, Sichuan, China.

[3]Department of Anesthesiology, Chengdu integrated TCM & Western Medicine Hospital, Chengdu, Sichuan, China.

*Corresponding author

[#] These authors contributed equally to this work

Emails of authors: 1172430737@qq.com (HQL); 814660661@qq.com (CHS); penglingjq@163.com (LP); weiw@scu.edu.cn (WW).

Abstract

Introduction: Postoperative delirium (POD) is a common complication after surgery. The association between sleep disorders and the risk of POD has been widely studied. Sleep disorders have emerged as a potential risk factor for POD, but recent studies provide conflicting evidence regarding the existence and the extent of the association. The aim of this systematic review and meta-analysis will be to estimate the association between sleep disorders and the risk of POD in adult patients.

Methods and analysis: A systematic review will be conducted to estimate the association between sleep disorders and the risk of POD in adult patients. This systematic review protocol follows the Preferred Reporting Items for Systematic Review and Meta-Analysis Protocols (PRISMA-P) statement. Literature searches will be carried out in the Pubmed, Embase, and Cochrane Library databases from inception until August 2025 without language restrictions. Only observational studies that investigated the association between sleep disorders and POD will be included. Two independent reviewers will be responsible for the selection of studies, extraction of information and risk of bias assessment of the articles. A random effects model will be used to calculate the pooled risk estimates for the outcome. Subgroup analysis will be used to explore potential sources of heterogeneity. Publication bias will be estimated by funnel plots and Egger's test. Sensitivity analysis will also be performed to explore the stability of the overall effect size. Also, evidence quality will be assessed. All data will be analysed using Review Manager (V.5.3) and Stata (V.14.0) statistical software.

Discussion: The proposed systematic review and meta-analysis will highlight the association of sleep disorders and the risk of POD in adult patients. The findings of this review

and meta-analysis will help clinicians consider the sleep disorders to make better health decisions.

Trial registration: Prospero registration number: CRD42024604118

Introduction

Postoperative delirium (POD) a is common and serious postoperative complication, affecting up to 50% of patients in some cohorts[1]. It is associated with a range of adverse outcomes, such as prolonged hospital stays, elevated healthcare costs, cognitive impairment, and higher risk of mortality and morbidity[2-4]. Given the significant adverse outcomes associated with POD, it is crucial for clinicians to identify risk factors and quantify their impact on POD.

Sleep is a complex physiological process that is essential for overall health and well-being. Sleep disorders are highly prevalent during the perioperative period, including conditions such as insomnia, sleep-disordered breathing, and circadian rhythm disruption. Recent studies have shown that 8.8%-79.1% of patients complained about poor sleep quality in the preoperative period[5-7]. Additionally, postoperative sleep disorders are highly prevalent in the first 2 weeks after surgery[8]. In the context of surgical patients, perioperative sleep disturbances may be associated with adverse prognostic outcomes, such as higher incidence of delirium and cognitive impairment, increased postoperative pain intensity, delayed postoperative recovery, increased hospital length of stay, and poor quality of life[9-11].

Extensive research has investigated the relationship between sleep disorders and the risk of POD. An earlier meta-analysis concluded that pre-operative sleep disturbances are likely associated with POD[12]. However, whether postoperative sleep disturbances serve as a risk factor for POD is still unknown. Initially, a meta-analysis conducted by Wang et al. had investigated association between perioperative sleep disturbance and the risk of POD[13]. Results showed that perioperative sleep disturbances were associated with an increased risk of POD in observational trials, yet no significant association was detected in randomized

controlled trials. Meanwhile, this study was limited by high heterogeneity and a small sample size, which may have affected the generalizability and statistical power of the findings. A recent meta-analysis incorporated 16 observational studies which concluded that sleep disturbance may increase the risk of POD[14]. This analysis offered valuable insights but was limited by relatively small sample sizes and the inclusion of only English-language literature.

However, since the publication of the previous meta-analysis, there have been additional studies published with large-scale investigations[15, 16]. Considering the importance of the topic, the aim of this study is to update the current evidence on the relationship between sleep disorders and the risk of POD.

Methods

Protocol and registration

This systematic review and meta-analysis was performed in accordance with the Preferred Reporting Items for Systematic Reviews and Meta-Analyses (PRISMA) guidelines[17] and the study protocol was registered in the International Prospective Register of Systematic Reviews (PROSPERO) (registration number: CRD42024604118). The PRISMA-P-checklist is shown in S1 File.

Ethics and dissemination

As this systematic review is summary and analysis of existing literature, there is no requirement for ethics approval. The results of the study will be published in a peer-reviewed journal.

Patient and public involvement

Patients and/or the public were not involved in the design, or conduct, or reporting, or dissemination plans of this research.

Objectives and outcomes

The sole objective of this is to determine whether sleep disorders are associated with an increased risk of POD in adult patients after surgery. The study aims to answer the research questions based on PECOS (Population, Exposure, Compares, Outcomes and Study design) criteria:

What is the association between sleep disorders and the risk of POD in adult patients after surgery?

PECOS eligibility criteria

- Population: adult patients aged 18 years and older undergoing surgery without pre-existing delirium/neurocognitive disorders before admission.
- Exposure: studies reporting on sleep disorders as exposure variables, defined as those occurring in either the pre- or post-operative period prior to delirium.
- Comparator: the population without exposure to sleep disorders served as a comparator.
- Outcome: the occurrence of POD measured by a validated scale, such as the 5th edition of Diagnostic and Statistical Manual of Mental Disorders (DSM-5), the Confusion Assessment Method (CAM), or Confusion Assessment Method for the Intensive Care Unit (CAM-ICU).
- Study design: We will only include observational studies (retrospective or prospective cohort studies, or case-control studies).

The inclusion criteria are as follows:

1. Studies investigating the association of sleep disorders and the risk of POD.
2. Included participants diagnosed with sleep disorders and no sleep disorders. The included

studies should report the subjectively or objectively diagnostic criteria for sleep disorders. Subjectively measured sleep disorders will be considered self-reported or obtained by any questionnaires (e.g., Pittsburgh Sleep Quality Index). Data from polysomnography (PSG) or actigraphy will be considered for objective measures of sleep disorders.

The exclusion criteria are as follows:

1. Studies without measurement of sleep disorders or POD.
2. Randomized controlled trials (RCTs), cross-sectional studies, review, meta-analyses, letter, editorial, protocol, case reports, or animal experiments.

Information sources

We will conduct searches in the following databases, including PubMed, Embase, CINAHL, and PsycINFO, for studies published before August 2025, that reported on the association between sleep disorders and postoperative delirium in adult patients. No restrictions will be imposed on language, thereby ensuring the inclusion of all potentially relevant studies. Additional studies will be identified by scanning the reference lists of the eligible studies.

Search strategy

The employed search keywords will include variations related to sleep, such as: ‘sleep’, ‘sleep initiation and maintenance disorders’, ‘sleep wake disorders’, and ‘sleep quality’, combined with terms associated with POD, including ‘postoperative delirium’, ‘delirium’, ‘emergence agitation’, ‘confusion’, ‘postoperative’, ‘surgery’ and ‘anesthesia’. Only observational studies, including cohort or case-control designs, will be included. The draft of the search strategy for each database is listed in S2 File.

Study selection

The records retrieved from the database search will be deduplicated in The Endnote X9 software reference manager. Then, two reviewers (QLH and CHS) will independently review the titles and abstracts according to the eligibility criteria. Full-text articles of the remaining records will be assessed against the eligibility criteria. Any disagreements will be resolved by consensus. The reasons for excluding full-text studies will be recorded and reported.

Data extraction

Two independent reviewers will extract the data from included studies using a Microsoft Excel spreadsheet. Any disagreements in data extraction will be resolved by discussion or consultation with a third reviewer. The following information will be collected: study characteristics (first author, publication year, age, country, number of patients, study design, type of surgery), sleep quality assessment details (sleep quality assessment tool, the timing of sleep disorder assessment, type of sleep disorder assessed, number of patients with good and poor sleep quality), delirium assessment details (delirium assessment tool, number of patients with and without delirium). In cases of missing or unclear data, the corresponding authors will be contacted for obtain the relevant information. If the number of the case group and the control group are not available by literature or mail, the study will be excluded.

Quality assessment

The Newcastle Ottawa Scale (NOS) assessment scale will be used to evaluate the quality of the included studies[18]. This assessment tool covers three domains: selection, comparability, and outcome or exposure, with score ranges from 0 to 9. High quality researches will be defined as seven or more scores, moderate quality as five to six scores and low quality as less than five. Any disagreements will be resolved by consensus.

Publication bias

Publication bias will be visually evaluated by the funnel plot. Any asymmetry detected in funnel plots will imply the presence of bias. Additionally, Egger's tests will be used to assess the severity of publication bias, with $P<0.05$ considered statistically significant.

Certainty of evidence

Two independent reviewers (QLH and CHS) will assess the meta-analysis results using the Grading of Recommendations, Assessment, Development and Evaluation (GRADE) approach[19]. We will summarize the quality of evidence across five dimensions, including risk of bias, indirectness of evidence, inconsistency in results, imprecision, and potential publication biases. According to GRADE, the quality of evidence can be qualified in one of four levels: high, moderate, low or very low. Any disagreements will be resolved through discussion or by consulting a third reviewer.

Data synthesis

In cases where two or more studies are available, reporting on the same outcome measures, a qualitative synthesis will be used to summarize the results of all included studies, otherwise narrative synthesis will be taken.

The meta-analysis will be conducted by RevMan 5.3 (Cochrane collaboration, Oxford, UK) and Stata 14(Stata Corp, College Station, TX, USA), considering a two-sided and p value less than 0.05 as statistically significant. The association between sleep disorders and POD will be evaluated by calculating the pooled odds ratios and associated 95% confidence intervals. The degree of heterogeneity was evaluated based on the Cochran’s Q statistics and I^2 statistics; a value greater than 50% and $p<0.05$ indicated substantial heterogeneity between studies. Owing

to differences in study design and settings, we used the random-effects model[20] to pool all odds ratios. If a study qualifies for inclusion in the systematic review but doesn't supply enough data for the meta-analysis, we'll also perform a systematic review along with a descriptive analysis.

When substantial heterogeneity is detected, we will investigate sources of heterogeneity by using meta-regression analysis or subgroup analysis. Subgroup analysis will be stratified by variables, such as study design (retrospective, prospective), mean population age (greater than 65, less than 65), sleep disorder type (i.e., insomnia, poor sleep quality, obstructive sleep apnoea, and unspecified sleep disorder), timing of sleep disorder (preoperative, postoperative before delirium), study country (China, outside China), and type of surgery (cardiac surgery, non-cardiac surgery) to determine whether other factors affected the findings. To further explore the sources of heterogeneity, meta-regression analyses will also be conducted, variables such as sample size, publication year, and percentage of female participants will be taken into account.

Additionally, sensitivity analysis will also be used to test whether the pooled results are reliable. Sensitivity analyses will be conducted using the leave-one-out method to evaluate the influence of each study. Finally, the evidence quality will be assessed by the GRADE approach.

Discussion

Many studies have investigated the correlation between sleep disorders exposure and POD outcomes[5, 21]. Sleep disorders are prevalent and common during the surgery, and may increase the risk of POD. The objective of this study is to elucidate the association of sleep disorders and the risk of POD in adult patients. The findings of this review and meta-analysis

will provide important data for future studies on intervention or treatment. Also, according to the results of the review and meta-analysis, patients with POD may take into consideration the sleep disorders to make better health decisions.

Key limitations of this review need to be considered. First, the different assessment methods of sleep disorders and the different definitions of POD can lead to a higher risk of publication. Therefore, subgroup analysis and meta-regression should be used to explore the source of heterogeneity. Second, it may be hard to establish causal relationships because most of the studies are expected to be observational. The GRADE tool will serve as a reference in the compilation of the summary table for the included studies. In general, our findings will help to improve the outcomes of patients with POD and provide better guidance for patients undergoing surgery.

Supporting Information

S1 File. PRISMA-P 2015 Checklist.

(DOCX)

S2 File. Search strategies for included databases.

(DOCX)

Data availability

No datasets were generated or analyzed during the current study. All relevant data from this study will be made available when the study is completed and published.

Conflicts of Interest

The authors have declared that no competing interests exist.

Author Contributions

Qianli Huang- Conceptualization, Data curation, Formal analysis, Methodology, Software, Writing – original draft, Writing – review & editing

Changhui Shao- Conceptualization, Data curation, Software, Supervision, Writing – review & editing

Ling Peng- Formal analysis, Methodology, Writing – review & editing

Wei Wei- Formal analysis, Methodology, Writing – review & editing

References

1. Inouye SK, Westendorp RG, Saczynski JS. Delirium in elderly people. Lancet (London, England). 2014;383(9920):911-22.
2. Yan E, Veitch M, Saripella A, Alhamdah Y, Butris N, Tang-Wai DF, et al. Association between postoperative delirium and adverse outcomes in older surgical patients: A systematic review and meta-analysis. Journal of clinical anesthesia. 2023;90:111221.
3. Huang H, Li H, Zhang X, Shi G, Xu M, Ru X, et al. Association of postoperative delirium with cognitive outcomes: A meta-analysis. Journal of clinical anesthesia. 2021;75:110496.
4. Bai J, Liang Y, Zhang P, Liang X, He J, Wang J, et al. Association between postoperative delirium and mortality in elderly patients undergoing hip fractures surgery: a meta-analysis. Osteoporosis international : a journal established as result of cooperation between the European Foundation for Osteoporosis and the National Osteoporosis Foundation of the USA. 2020;31(2):317-26.
5. Butris N, Tang E, Pivetta B, He D, Saripella A, Yan E, et al. The prevalence and risk factors of sleep disturbances in surgical patients: A systematic review and meta-analysis. Sleep medicine reviews. 2023;69:101786.
6. Ida M, Onodera H, Yamauchi M, Kawaguchi M. Preoperative sleep disruption and postoperative functional disability in lung surgery patients: a prospective observational study. Journal of anesthesia. 2019;33(4):501-8.
7. Rhon DI, Snodgrass SJ, Cleland JA, Cook CE. Comorbid Insomnia and Sleep Apnea are Associated with Greater Downstream Health Care Utilization and Chronic Opioid Use after Arthroscopic Hip Surgery. Pain physician. 2019;22(4):E351-e60.
8. Wang Y, Jiang Y, Chen T, Xia Q, Wang X, Lv Q, et al. Prediction of risk factors of sleep disturbance in patients undergoing total hip arthroplasty. Sleep and biological rhythms. 2024;22(1):85-91.
9. Lam EWK, Chung F, Wong J. Sleep-Disordered Breathing, Postoperative Delirium, and Cognitive Impairment. Anesthesia and analgesia. 2017;124(5):1626-35.
10. Kjølhede P, Langström P, Nilsson P, Wodlin NB, Nilsson L. The impact of quality of sleep on recovery from fast-track abdominal hysterectomy. Journal of clinical sleep medicine : JCSM : official publication of the American Academy of Sleep Medicine. 2012;8(4):395-402.
11. Niklasson A, Finan PH, Smith MT, Forsberg A, Dietz N, Kander T, et al. The relationship

between preoperative sleep disturbance and acute postoperative pain control: A systematic review and meta-analysis. Sleep medicine reviews. 2024;79:102014.
12. Fadayomi AB, Ibala R, Bilotta F, Westover MB, Akeju O. A Systematic Review and Meta-Analysis Examining the Impact of Sleep Disturbance on Postoperative Delirium. Critical care medicine. 2018;46(12):e1204-e12.
13. Wang H, Zhang L, Zhang Z, Li Y, Luo Q, Yuan S, et al. Perioperative Sleep Disturbances and Postoperative Delirium in Adult Patients: A Systematic Review and Meta-Analysis of Clinical Trials. Frontiers in psychiatry. 2020;11:570362.
14. He E, Dong Y, Jia H, Yu L. Relationship of sleep disturbance and postoperative delirium: a systematic review and meta-analysis. Gland surgery. 2022;11(7):1192-203.
15. Huang Z, Huang C, Deng Y, Lu H, Shi L. Association Between Preoperative Long-Term Poor Sleep Quality and Postoperative Delirium in Elderly Patients Undergoing Cardiac Surgery: A Multi-Center Observational Study. The American surgeon. 2024;90(6):1260-7.
16. Ou-Yang CL, Ma LB, Wu XD, Ma YL, Liu YH, Tong L, et al. Association of sleep quality on the night of operative day with postoperative delirium in elderly patients: A prospective cohort study. European Journal of Anaesthesiology. 2024;41(3):226-33.
17. Page MJ, McKenzie JE, Bossuyt PM, Boutron I, Hoffmann TC, Mulrow CD, et al. The PRISMA 2020 statement: an updated guideline for reporting systematic reviews. BMJ (Clinical research ed). 2021;372:n71.
18. Stang A. Critical evaluation of the Newcastle-Ottawa scale for the assessment of the quality of nonrandomized studies in meta-analyses. European journal of epidemiology. 2010;25(9):603-5.
19. Guyatt GH, Oxman AD, Vist GE, Kunz R, Falck-Ytter Y, Alonso-Coello P, et al. GRADE: an emerging consensus on rating quality of evidence and strength of recommendations. BMJ (Clinical research ed). 2008;336(7650):924-6.
20. DerSimonian R, Laird N. Meta-analysis in clinical trials. Controlled clinical trials. 1986;7(3):177-88.
21. Lin D, Huang X, Sun Y, Wei C, Wu A. Perioperative Sleep Disorder: A Review. Frontiers in medicine. 2021;8:640416.

Dear Editor and Reviewers

Thank you very much for giving us the opportunity to revise our manuscript (PONE-D-25-29095). We sincerely appreciate the valuable comments and suggestions provided by the reviewers, which have significantly helped us to improve the quality of our manuscript. We have addressed each of the reviewers' comments in detail below.

Journal requirements:

1. Please ensure that your manuscript meets PLOS ONE's style requirements, including those for file naming.

Reply: We have carefully checked throughout the manuscript.

2. Thank you for stating the following financial disclosure:

[The research work was funded by a grant from the National Natural Science Foundation of China (grant no. 81971772).].

Please state what role the funders took in the study. If the funders had no role, please state: "The funders had no role in study design, data collection and analysis, decision to publish, or preparation of the manuscript."

If this statement is not correct you must amend it as needed.

Please include this amended Role of Funder statement in your cover letter; we will change the online submission form on your behalf.

Reply: The funders had no role in study design, data collection and analysis, decision to publish, or preparation of the manuscript.

3. Please remove any funding-related text from the manuscript and let us know how you would like to update your Funding Statement. Currently, your Funding Statement reads as follows:

[The research work was funded by a grant from the National Natural Science Foundation of China (grant no. 81971772).]

Please include your amended statements within your cover letter; we will change the online submission form on your behalf.

Reply: we have removed any funding-related text from the manuscript and included our amended statements within cover letter.

Comments to the Author

Reviewer #1:

1. Pg 3: Financial disclosure statement missing

Reply: Thank you for your suggestion. We have added the financial disclosure statement.

2. Pg 6 ln 120-121: ? does this include pre, peri and post op period? or just the perioperative period? Please clarify as earlier on you are presenting data from all periods?

Reply: We have modified the description of the perioperative sleep disorders. The timing of sleep disorders includes either the pre- or post-operative period prior to delirium.

3. Pg 6 ln 123: ?This is not an outcome. You need to define what outcomes you are specifically measuring - e.g. incidence of POD

Reply: Thank you very much for your valuable comments, we have revised the outcomes.

4. Pg 6 ln 124-125: If you are looking for an association between the exposure and outcome, then why are you including cross sectional studies? Also, you discussed conflicts within

literature between observational studies and RCT, yet you are excluding RCT - please justify your reasoning behind this.

Reply: Thank you very much for pointing out this issue. Given that cross-sectional studies cannot determine the association between the exposure and outcome, we have excluded the cross-sectional studies. In recent years, several observational studies have explored the association between sleep disorders and POD; however, few newly published RCTs have addressed this topic, so this review could not provide new insights into the association between sleep disorders and POD based on RCTs.

5. Pg 6 ln 128-19: the use of insomnia symptoms or poor sleep quality are subjective measures, and thus if you are going to include them you need to clearly define what is meant by these. Also, how is poor sleep quality being identified? Using well defined criteria/diagnoses would provide a more objective and standard/consistent measure for sleep disorders.

Reply: Thank you very much for your valuable comments. We have provided a more objective and standard/consistent measure for sleep disorders.

6. Pg 6 ln 132: Also RCT according to your earlier criteria.

Reply: Thank you for your suggestions. We have added this information in the exclusion criteria.

7. Pg 7 ln" 134: I am curious why Embase is being used, given its primary focus is on pharmaceuticals/drugs?

What about alternative databases, like CINAHL, especially as you indicate you are only including observational studies?

Reply: Thank you for your suggestions. We have added the CINAHL database for a comprehensive search strategy according to the suggested idea.

8. Pg 16 ln 194-199: what about the pre-existence of cognitive impairment/dementia, which is an important influencing factor, that can also impact on the diagnosis of POD?

Reply: Thank you very much for this good question. We have revised the participants of inclusion criteria.

Reviewer #2:

1. I have some concerns about the methodology you propose to study this topic. You indicate that your search will be limited to observational studies; however, you plan to search Cochrane Library, which indexes systematic reviews (CDSR) and randomized controlled trials (Central). This seems contradictory. Similarly, you justify your study by noting that since the publication of prior reviews there have been additional studies published which may contribute to further understanding; however, both of the additional studies cited appear to concern preoperative sleep disturbances, not postoperative.

Reply: Thank you very much for this good question. We have deleted the Cochrane Library. We have added this information in the manuscript as “However, since the publication of the previous meta-analysis, there have been additional studies published with large-scale investigations[15, 16]. Considering the importance of the topic, the aim of this study is to update the current evidence on the relationship between sleep disorders and the risk of POD.” The cited study, "Association of sleep quality on the night of the operative day with postoperative delirium in elderly patients: A prospective cohort study," explored the association between postoperative sleep disturbances and POD.

2. Your search strategy also has issues that limit the methodological quality of the proposed review. You are missing potential synonyms such as emergence agitation and post surgical

(with space). There are also unexplained differences in free-text search terms between different databases. Suggest also considering a search of PsycINFO, given the topic area.

Reply: Thank you for pointing this out. We have added keywords such as "emergence agitation" and "post-surgical," as well as the PsycINFO database.

In summary, we have incorporated all the reviewers' suggestions to the best of our ability, and we believe the revised manuscript has been significantly improved as a result. A detailed list of changes is highlighted in the revised manuscript.
