# Supplementary material for: The relationship between sleep disorders and postoperative delirium in adult patients: Protocol for an updated systematic review and meta-analysis
Source: PLoS One. 2025 Dec 2;20(12):e0337598. doi: 10.1371/journal.pone.0337598 (PMC12671798; doi:10.1371/journal.pone.0337598)
Supplement: Attachment — Submitted filename: Response to Reviewers.docx [file pone.0337598.s004.docx]

**Dear Editor and Reviewers**

Thank you very much for giving us the opportunity to revise our manuscript (PONE-D-25-29095). We sincerely appreciate the valuable comments and suggestions provided by the reviewers, which have significantly helped us to improve the quality of our manuscript. We have addressed each of the reviewers’ comments in detail below.

**Journal requirements:**

1. Please ensure that your manuscript meets PLOS ONE's style requirements, including those for file naming.

Reply: We have carefully checked throughout the manuscript.

2. Thank you for stating the following financial disclosure:

[The research work was funded by a grant from the National Natural Science Foundation of China (grant no. 81971772).].

Please state what role the funders took in the study.  If the funders had no role, please state: "The funders had no role in study design, data collection and analysis, decision to publish, or preparation of the manuscript."

If this statement is not correct you must amend it as needed.

Please include this amended Role of Funder statement in your cover letter; we will change the online submission form on your behalf.

Reply: The funders had no role in study design, data collection and analysis, decision to publish, or preparation of the manuscript.

3. Please remove any funding-related text from the manuscript and let us know how you would like to update your Funding Statement. Currently, your Funding Statement reads as follows:

[The research work was funded by a grant from the National Natural Science Foundation of China (grant no. 81971772).]

Please include your amended statements within your cover letter; we will change the online submission form on your behalf.

Reply: we have removed any funding-related text from the manuscript and included our amended statements within cover letter.

**Comments to the Author**

Reviewer #1: 
1. Pg 3: Financial disclosure statement missing

Reply: Thank you for your suggestion. We have added the financial disclosure statement.

2. Pg 6 ln 120-121: ? does this include pre, peri and post op period? or just the perioperative period? Please clarify as earlier on you are presenting data from all periods?

Reply: We have modified the description of the perioperative sleep disorders. The timing of sleep disorders includes either the pre- or post-operative period prior to delirium.

3. Pg 6 ln 123: ?This is not an outcome. You need to define what outcomes you are specifically measuring - e.g. incidence of POD

Reply: Thank you very much for your valuable comments, we have revised the outcomes.

4. Pg 6 ln 124-125: If you are looking for an association between the exposure and outcome, then why are you including cross sectional studies? Also, you discussed conflicts within literature between observational studies and RCT, yet you are excluding RCT - please justify your reasoning behind this.

Reply: Thank you very much for pointing out this issue. Given that cross-sectional studies cannot determine the association between the exposure and outcome, we have excluded the cross-sectional studies. In recent years, several observational studies have explored the association between sleep disorders and POD; however, few newly published RCTs have addressed this topic, so this review could not provide new insights into the association between sleep disorders and POD based on RCTs.

5. Pg 6 ln 128-19: the use of insomnia symptoms or poor sleep quality are subjective measures, and thus if you are going to include them you need to clearly define what is meant by these. Also, how is poor sleep quality being identified? Using well defined criteria/diagnoses would provide a more objective and standard/consistent measure for sleep disorders.

Reply: Thank you very much for your valuable comments. We have provided a more objective and standard/consistent measure for sleep disorders.

6. Pg 6 ln 132: Also RCT according to your earlier criteria.

Reply: Thank you for your suggestions. We have added this information in the exclusion criteria.

7. Pg 7 ln" 134: I am curious why Embase is being used, given its primary focus is on pharmaceuticals/drugs?
What about alternative databases, like CINAHL, especially as you indicate you are only including observational studies?

Reply: Thank you for your suggestions. We have added the CINAHL database for a comprehensive search strategy according to the suggested idea.
8. Pg 16 ln 194-199: what about the pre-existence of cognitive impairment/dementia, which is an important influencing factor, that can also impact on the diagnosis of POD?

Reply: Thank you very much for this good question. We have revised the participants of inclusion criteria.
**Reviewer #2:**

1. I have some concerns about the methodology you propose to study this topic. You indicate that your search will be limited to observational studies; however, you plan to search Cochrane Library, which indexes systematic reviews (CDSR) and randomized controlled trials (Central). This seems contradictory. Similarly, you justify your study by noting that since the publication of prior reviews there have been additional studies published which may contribute to further understanding; however, both of the additional studies cited appear to concern preoperative sleep disturbances, not postoperative.
Reply: Thank you very much for this good question. We have deleted the Cochrane Library. We have added this information in the manuscript as “However, since the publication of the previous meta-analysis, there have been additional studies published with large-scale investigations[15, 16]. Considering the importance of the topic, the aim of this study is to update the current evidence on the relationship between sleep disorders and the risk of POD.” The cited study, "Association of sleep quality on the night of the operative day with postoperative delirium in elderly patients: A prospective cohort study," explored the association between postoperative sleep disturbances and POD.

2. Your search strategy also has issues that limit the methodological quality of the proposed review. You are missing potential synonyms such as emergence agitation and post surgical (with space). There are also unexplained differences in free-text search terms between different databases. Suggest also considering a search of PsycINFO, given the topic area.

Reply: Thank you for pointing this out. We have added keywords such as "emergence agitation" and "post-surgical," as well as the PsycINFO database.

In summary, we have incorporated all the reviewers’ suggestions to the best of our ability, and we believe the revised manuscript has been significantly improved as a result. A detailed list of changes is highlighted in the revised manuscript.
