# Supplementary material for: The relationship between sleep disorders and postoperative delirium in adult patients: Protocol for an updated systematic review and meta-analysis
Source: PLoS One. 2025 Dec 2;20(12):e0337598. doi: 10.1371/journal.pone.0337598 (PMC12671798; doi:10.1371/journal.pone.0337598)
Supplement: Attachment — Submitted filename: Response_to_Reviewers_auresp_2.docx [file pone.0337598.s006.docx]

**Dear Editor and Reviewers:**

We would like to thank the Editors and Reviewers for their thoughtful and constructive comments. We have addressed each point below. All changes are indicated in the tracked version of the revised manuscript and are described below in a point-by-point response.

Suggestions for further improvement:
1. The addition of the keyword "emergence agitation" and “post surgical” in the search strings of the various databases is a positive step. In the Search Strategy paragraph, I recommend also including the term "post surgical," as suggested by Reviewer 2.

Response: Thank you for your suggestion. We have added the term “post surgical” as suggested.
2. Furthermore, I believe it would be useful to specify the use of Boolean operators (AND, OR) in the Search Strategy paragraph as well, in order to clarify how the different groups of keywords are connected in the search.

Response: We have added Boolean operators (“AND” and “OR”) in the Search Strategy paragraph to combine search terms.

3. Minor grammatical and syntactic improvements:
•Line 33: “Pubmed” → “PubMed”

Response: We appreciate your careful reading of our manuscript. We have revised as suggested.
• Line 39: “used” → “conducted”

Response: We have revised as suggested.
• Line 68: “a” after “is,” not before (typographical error)

Response: We have revised as suggested.
• Line 86: “had investigated association” → “had investigated the association” (add “the”)

Response: We have added “the” as suggested.
• Line 106: “is summary and analysis” → “is a summary and analysis” (missing article)

Response: We have added “a” as suggested.
• Line 112: “The sole objective of this is to determine” → “The sole objective of this study is to determine” (add “study” for clarity)

Response: We have added “study” as suggested.
• Line 114: “Compares” → In the PECOS model, C stands for “Comparator,” not “Compares”

Response: We have revised as suggested.
• Line 132: “association of sleep disorders and the risk of POD” → “association between sleep disorders and the risk of POD”

Response: We have revised as suggested.
• Lines 132-133: “Included participants diagnosed with sleep disorders and no sleep disorders.” → “Studies including participants with and without diagnosed sleep disorders.”

Response: We have revised as suggested.
• Line 133: “subjectively or objectively diagnostic criteria” → “subjective or objective diagnostic criteria”

Response: We have revised as suggested.
• Line 143: Remove comma after “2025”

Response: We have revised as suggested.
• Line 155: “in The Endnote X9 software reference manager” → “in EndNote X9 reference management software”

Response: We have revised as suggested.
• Line 169: “contacted for obtain the relevant information” → “contacted to obtain the relevant information”

Response: We have revised as suggested.
• Line 173: Avoid repeating “scale” twice in “The Newcastle Ottawa Scale (NOS) assessment scale will be used…”

Response: We have revised as suggested.
• Line 174: “researches” → “studies” (in scientific English “research” is uncountable, use “studies or research studies” in plural)

Response: We have revised as suggested.
• Line 192: “otherwise narrative synthesis will be taken” → “otherwise a narrative synthesis will be carried out”

Response: We have revised as suggested.
• Lines 197: “was evaluated” → Should be future tense: “will be evaluated”

Response: We have revised as suggested.
• Lines 217-219: “Sleep disorders are prevalent and common during the surgery” → I think that “during the perioperative period” is more appropriate

Response: We have revised as suggested.
• Lines 219-220: “association of sleep disorders and the risk of POD” → “association between sleep disorders and the risk of POD”

Response: We have revised as suggested.
• Lines 222-223: “patients with POD may take into consideration the sleep disorders to make better health decisions” → “patients with POD may take sleep disorders into account when making health decisions”

Response: We have revised as suggested.
• Line 225: “higher risk of publication” → Add “bias”: “higher risk of publication bias”

Response: We have added “bias” as suggested.
• Lines 227: “should be used to explore the source of heterogeneity” → “will be used to explore sources of heterogeneity”

Response: We have revised as suggested.

4. Overall recommendation:
The manuscript shows substantial enhancement and better alignment with PLOS ONE’s publication criteria. Pending minor revisions as outlined, I believe it merits acceptance for publication. The authors are encouraged to refine the indicated sections to further improve transparency and reproducibility.

Response: We are truly grateful for your thoughtful evaluation and constructive feedback, which have greatly contributed to the improvement of our manuscript.
